# Br Vacancy Defects Healed Perovskite Indoor Photovoltaic Modules with Certified Power Conversion Efficiency Exceeding 36%

**DOI:** 10.1002/advs.202204138

**Published:** 2022-10-17

**Authors:** Cuiling Zhang, Chong Liu, Yanyan Gao, Shusheng Zhu, Fang Chen, Boyuan Huang, Yi Xie, Yaqing Liu, Mengen Ma, Zhen Wang, Shaohang Wu, Ruud E. I. Schropp, Yaohua Mai

**Affiliations:** ^1^ Institute of New Energy Technology College of Information Science and Technology Guangdong Engineering Research Center of Thin‐Film Photovoltaic Processes and Equipment and Key Laboratory of New Semiconductors and Devices of Guangdong Higher Education Institutes Jinan University Guangzhou 510632 China; ^2^ Department of Materials Science and Engineering Southern University of Science and Technology Shenzhen Guangdong 518055 China; ^3^ Institute for Advanced Materials and Guangdong Provincial Key Laboratory of Optical Information Materials and Technology South China Academy of Advanced Optoelectronics South China Normal University Guangzhou 510006 China

**Keywords:** Br vacancy defect, indoor photovoltaic cells, module, wide‐bandgap perovskites

## Abstract

Indoor photovoltaics (IPVs) are expected to power the Internet of Things ecosystem, which is attracting ever‐increasing attention as part of the rapidly developing distributed communications and electronics technology. The power conversion efficiency of IPVs strongly depends on the match between typical indoor light spectra and the band gap of the light absorbing layer. Therefore, band‐gap tunable materials, such as metal‐halide perovskites, are specifically promising candidates for approaching the indoor illumination efficiency limit of ∼56%. However, perovskite materials with ideal band gap for indoor application generally contain high bromine (Br) contents, causing inferior open‐circuit voltage (*V*_OC_). By fabricating a series of wide‐bandgap perovskites (Cs_0.17_FA_0.83_PbI_3−_ *_x_* Br*_x_*, 0.6 ≤ *x* ≤ 1.6) with varying Br contents and related band gaps, it is found that, the high Br vacancy (V_Br_) defect density is a significant reason that leading to large *V*_OC_ deficits apart from the well‐accepted halide segregation. The introduction of I‐rich alkali metal small‐molecule compounds is demonstrated to suppress the V_Br_ and increase the *V*_OC_ of perovskite IPVs up to 1.05 V under 1000 lux light‐emitting diode illumination, one of the highest *V*_OC_ values reported so far. More importantly, the modules are sent for independent certification and have gained a record efficiency of 36.36%.

## Graphical abstract

The V_Br_ are easy to form in wide‐bandgap perovskite films, and the introduction of I‐rich alkali metal small‐molecule compounds is demonstrated to heal the V_Br_ defects and increase the *V*_OC_ of perovskite indoor photovoltaic cells. In this work, a stable perovskite indoor photovoltaic module with an independent certified efficiency of 36.36% at 1000 lux TL84 illumination is also reported.

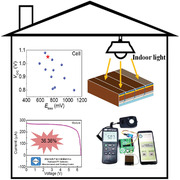

## 1 Introduction

The Internet of Things (IoT) has made impressive progress, especially in low‐power wide‐area network technology.^[^ ^1^ ^]^ The power consumption of some protocols on certain chips is as low as in the µW range, comparable to the power of incident light on less than a square centimeter under normal indoor light illumination conditions.^[^ ^2^ ^]^ Thus, typically available indoor light energy is considered to be very well capable of powering IoT devices using tailored indoor photovoltaics (IPVs). Different from outdoor sunlight, the spectra of indoor artificial light sources (e.g., light emitting diode (LED), fluorescent lamp (FL), etc.) are generally limited to the visible region (380–780 nm). As a result, the ideal band gap for IPVs ranges from 1.7 to 1.9 eV and yield an efficiency limit that as high as ∼56%.^[^ ^3^ ^]^ In addition, the light illuminance is generally 200–1000 lux in an indoor environment, corresponding to a light intensity of 0.18–0.90 mW cm^−2^.^[^ ^4^ ^]^ Since the photocurrent is rather low, IPV devices are insensitive to series resistance (*R*_s_) due to the small electrical voltage drop on the series element, while the shunt resistance (*R*_sh_) plays an important role as shunting current may be comparable to the photocurrent.^[^ ^5^ ^]^ Owing to the influence of spectrum and photocurrent, the commonly used photovoltaic devices (e.g., silicon, cadmium telluride (CdTe), copper indium gallium selenide (CIGS) solar cells) generally have a poor performance under indoor light source illumination.

Fortunately, recent emerging halide perovskite IPVs have been reported to achieve high efficiency over 41% for small area (0.14 cm^2^) device,^[^ ^6^ ^]^ though standardized indoor efficiency evaluation methods are still under development.^[^ ^7^ ^]^ High bromine (Br) substitution has generally been used to widen the band gap of perovskite absorber materials to the ideal range of IPVs, but accompanied by the halide separation issue simultaneously.^[^ ^8^ ^]^ Many efforts, including alloying cations in the perovskite lattice,^[^ ^9^ ^]^ controlling the crystal size,^[^ ^10^ ^]^ developing dopant‐free hole transport material,^[^ ^11^ ^]^ and surface passivation,^[^ ^12^ ^]^ have been introduced to restrict halide segregation in Br‐rich perovskite films. For example, Gao et al. discovered that halide separation can be highly suppressed by embedding nanocrystals of mixed‐halide perovskites in an endotaxial matrix.^[^ ^8^ ^]^ Brabec and co‐authors demonstrated that the strain‐activated halide segregation could be suppressed via release the strain by additive of potassium iodide (KI).^[^ ^13^ ^]^ However, the Br vacancy (V_Br_) defects should be concerned in addition to halide separation for Br‐rich wide‐bandgap devices, as the Br can easily escape from film surface due to its higher vapor pressure than iodine (I).^[^ ^14^, ^15^ ^]^ Indeed, the V_Br_ defects have been demonstrated to be the one of the dominant barriers to achieve high‐efficiency Br‐rich perovskite outdoor solar cells.^[^ ^16^, ^17^ ^]^ Wang et al. have calculated the formation energies of V_Br_, showing that the formation energy of the dominant defect under Br‐rich growth condition is much lower than that under moderate or Br‐poor conditions.^[^ ^18^ ^]^ However, study on the importance of vacancy defects to the performance of IPVs is still lacking. As mentioned above, the *R*_sh_ shows considerable effect on the performance of IPVs.^[^ ^19^ ^]^ The vacancy defects in perovskite film provide nonradiative recombination centers and aggravate the ion migration, forming leakage channels that significantly affect the open‐circuit voltage (*V*_OC_) of photovoltaic cells under weak illumination.^[^ ^17^ ^]^ Moreover, the efficiency of state‐of‐the‐art perovskite indoor photovoltaic module is falling far behind that of small area IPVs thus far, only reaching 17.89% (area = 4 cm^2^).^[^ ^20^ ^]^ In addition, although the notorious instability issue of perovskite IPVs can be weakened as the more moderate operation environment, intrinsically stable methylamine (MA)‐free perovskite is assumed to be more promising for IPVs products with long lifetime.^[^ ^21^, ^22^, ^23^ ^]^

In this work, band‐gap tunable I/Br hybrid MA‐free perovskite IPVs were fabricated in order to study the correlation between the band gap and the photovoltaic performance. Subsequently, we introduced I‐rich alkali metal small‐molecule material KI treatment that effectively addressed the V_Br_ issue. As a result, the champion perovskite indoor photovoltaic module gained a certified record power conversion efficiency (PCE) of 36.36%, which has groundbreaking significance for the development of perovskite indoor photovoltaic field.

## 2 Results and Discussion

### 2.1 Accurate Measurement of IPVs

To obtain accurate evaluation of photovoltaic performance, confirming the incident illumination energy is one of the most important prerequisites. However, it is not an easy task when evaluating low‐powered indoor light. Parameters, such as illuminance, color rendering index, and color temperature that are concerned in lighting and display have a certain relationship with energy intensity and spectra, which usually vary greatly from supplier to supplier. For instance, the emission spectrum of an FL is different from that of an LED, and even light sources of the same type operating at different color temperature have different emission spectra (Figure S1, Supporting Information). The limit of photovoltaic parameters is directly affected by the spectrum according to the Shockley–Queisser (S‐Q) formula (Figures S2–S5 and Note S1, Supporting Information). Recently, Hou et al. systematically studied the origins of the short‐circuit current density (*J*_SC_) measurement errors, including light intensity calibration, edge effect, and light source spatial homogeneity, etc.^[^ ^7^ ^]^ For accurate evaluation of IPVs, the calculation of theoretical limit of photovoltaic parameters, especially the *J*_SC_, is essential in accuracy estimation and error prevention.

### 2.2 Band Gap Tuning

**Figure 1:**
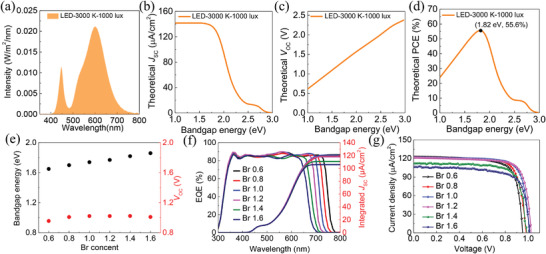
a) Illumination spectrum of a 3000 K color temperature LED at 1000 lux. The S‐Q limit of b) *J*_SC_, c) *V*_OC_, and d) PCE of an ideal photovoltaic device as a function of band‐gap energy under the 3000 K LED. e) Optical absorption band‐gap energy and *V*_OC_ of the Cs_0.17_FA_0.83_PbI_1−_ *_x_* Br*_x_* as a function of Br content. f) EQE curves and integrated *J*_SC_ of the devices with different Br contents. The integrated *J*_SC_ was calculated by integrating the EQE curve over the photon flux spectrum of the 3000 K LED. g) *J*–*V* curves of the devices with different Br contents measured under 3000 K LED (illuminance: 1000 lux, irradiance: 301.9 µW cm^−2^) illumination.

An LED (3000 K, 1000 lux) lamp is used for the indoor light source. Its spectrum is shown in **Figure** **1**a. Since the LED spectrum is limited to the range from 380 to 780 nm, a narrow‐bandgap absorber is unsuitable for application in IPVs as it would not lead to an increased *J*_SC_ while causing a reduced *V*_OC_. As the band gap decreases to more than 1.56 eV, the calculated *J*_SC_ approaches to the maximum value of 141.58 µA cm^−2^ (Figure 1b). The *V*_OC_ depends linearly on the band gap, as shown in Figure 1c. As a result, the theoretical optimal band gap of IPVs is 1.82 eV, allowing a theoretical efficiency upper‐limit of 55.6% after balancing the *J*_SC_ and the *V*_OC_ (Figure 1d). The detailed calculation method of photovoltaic parameters limit can be seen in Note S1 in the Supporting Information. To verify the above conclusion, we designed and then fabricated MA‐free perovskites with a series of I/Br ratios. Figure 1e displays the relationship between band‐gap energies of Cs_0.17_FA_0.83_PbI_3−_ *_x_* Br*_x_* (*x* = 0.6, 0.8, 1.0, 1.2, 1.4, 1.6) perovskite materials and their Br content. The band‐gap values were obtained by fitting the long‐wavelength cutoff edges in the external quantum efficiency (EQE) curves (Figure 1f and Table S1, Supporting Information).^[^ ^24^ ^]^ The band gap enlarged linearly with ever‐increasing Br content, which is consistent with previous report.^[^ ^25^ ^]^ For simplicity, we denote the perovskite films and devices as Br 0.6, Br 0.8, Br 1.0, Br 1.2, Br 1.4, and Br 1.6. The fabrication process of the perovskite IPVs follows our previously reported inverted planar structure process: indium tin oxide (ITO)/poly[bis(4‐phenyl) (2,4,6‐trimethylphenyl) amine] (PTAA)/Al_2_O_3_/perovskite/[6,6]‐phenyl‐C61‐butyric acid methyl ester (PCBM)/bathocuproine (BCP)/Ag (Figure S6, Supporting Information).^[^ ^26^ ^]^ Figure S7 in the Supporting Information displays the photovoltaic merits of the as‐fabricated perovskite IPVs with varying Br contents. The *J*_SC_ drops slightly before the Br content (*x*) increases to 1.2, and drops rapidly as further increase. This is ascribed to the steep decrease in photon absorption as the wavelength is greater than 600 nm. Unfortunately, the *V*_OC_ does not increase consistently when Br contents increase from 1.0 to 1.6 (Figure 1e and Figures S7–S9, Supporting Information), which may be attributed to the severe nonradiative recombination.^[^ ^27^ ^]^ Typical reverse‐scan *J*–*V* curves are exhibited in Figure 1g and a champion efficiency of 32.61%, with a *J*_SC_ of 121.1 µA cm^−2^, a *V*_OC_ of 1.02 V, and a fill factor (FF) of 79.7% is obtained for sample Br 1.2 at 1000 lux. The integrated *J*_SC_ calculated from the EQE spectra is well matched with the value obtained from the *J*–*V* measurements (deviation less than 5%), which confirms that our *J*_SC_ is not overestimated. This can be attributed to great effort in maximizing the accuracy of the measurements for IPVs, including a darkroom installation with minimal stray light, a light box homogenizing plate, light intensity calibration using 3D positioning of the test fixture.

The severe nonradiative recombination present in the Br‐rich perovskite thin films is partly ascribed to halide segregation,^[^ ^13^, ^28^ ^]^ as evidenced by the measured splitting of photoluminescence (PL) spectra (Figure S10, Supporting Information). The PL emission peak moves toward short wavelength and starts to split along with the gradually increasing Br content. Two perovskite materials with different band gaps are identified as Br‐ and I‐rich perovskites.^[^ ^8^ ^]^ As shown in Figure S11 in the Supporting Information, the Br 1.2 film shows slight halide segregation while the *V*_OC_ loss of the perovskite photovoltaic cell is significantly increased, which indicates that the *V*_OC_ deficit is also dominated by other reasons apart from halide segregation. According to the literature, charge state of the vacancy also significantly accelerates the electron–hole recombination, which is assumed to be responsible for the additional *V*_OC_ loss. The *V*_OC_ under standard outdoor 1‐sun irradiation (AM1.5 G, 100 mW cm^−2^) shows a similar trend to that under indoor light while the *J*_SC_ changes linearly, leading to a different optimal Br content, namely, Br 0.8 (Figures S12 and S13, Supporting Information). Therefore, photovoltaic cells exhibiting high efficiency under sunlight cannot ensure equally excellent performance under indoor illumination. This novel finding will provide guidance to optimize the IPVs separately rather than as an additional application for optimizing outdoor solar cells.

**Figure 2:**
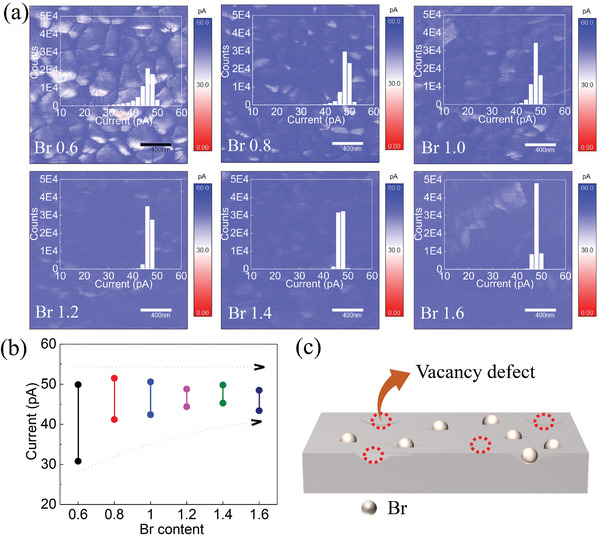
a) c‐AFM images and quantized photocurrent statistics of Cs_0.17_FA_0.83_PbI_3−_ *_x_* Br*_x_* films with different Br contents. b) Photocurrent analysis of the perovskite films fabricated with different Br contents. c) Schematic diagram of vacancy defect formation.

In order to verify and quantify the V_Br_, conductive atomic force microscopy (c‐AFM) measurements were performed to characterize the photocurrent distribution over the perovskite films surface.^[^ ^29^, ^30^ ^]^ The samples were irradiated by an LED light source with an intensity of 15 µW cm^−2^ from the ITO side. It can be seen that the current is distributed nonuniformly over the grain boundaries and surfaces of Br 0.6‐based perovskite thin film, where the current signals in grain boundaries are much higher than that on grain surfaces (**Figure** **2**a). This has been explained by the accumulation of charged ion defects at the grain boundaries, making the grain boundaries more conductive.^[^ ^31^ ^]^ In comparison, the differences in current signals between grain surfaces and boundaries are drastically reduced in Br‐rich films. The quantized current range is shown in Figure 2b, the maximum value of current (∼50 pA) is basically the same, while the minimum value of current consistently increases from ∼30 to ∼45 pA with increasing Br content, indicating the growing leakage current at grain surfaces. It has been reported that the Cs/Br‐based wide‐bandgap perovskite films are prone to have high defect densities, due to the highly accelerated dynamic speed of perovskite crystallization.^[^ ^32^ ^]^ In addition, the Br possesses high vapor pressure and escapes easily from the perovskite grain surfaces during the annealing process.^[^ ^14^, ^15^ ^]^ The V_Br_ are charged and they accumulate on the grain surfaces, resulting in the observed increase of conductivity (Figure 2c). The V_Br_ defect density correlates positively with the Br content. Therefore, the effect of V_Br_ on nonradiative recombination in Br‐rich perovskite IPVs cannot be ignored.

In addition, the formation energies (*E*_form_) of V_Br_ in CsPbI_2.4_Br_0.6_, CsPbI_1.8_Br_1.2_ and CsPbI_1.4_Br_1.6_ materials were calculated using the first‐principles density functional theory (DFT). Note that Cs^+^ is used as the A site cation to simplify the modeling. As shown in **Table** **1**, *E*_form_ decreases from 2.84 eV (CsPbI_2.4_Br_0.6_) to 2.51 eV (CsPbI_1.4_Br_1.6_), indicating that Br vacancies are more easily formed in Br‐rich samples. This is consistent with the literature results reporting that the *E*_form_ of the dominant V_Br_ defect in CsPbBr_3_ perovskite is rather low.^[^ ^18^ ^]^

**Table 1:** Calculated formation energies for vacancy defects in CsPbI_3−_ *_x_* Br*_x_* lattice

Br content	*E* (vacancy)	*E* (supercell)	*µ* (atom)	*E*_form_
CsPbI_2.4_Br_0.6_	−285.20 eV	−289.54 eV	−1.50 eV	2.84 eV
CsPbI_1.8_Br_1.2_	−294.22 eV	−298.34 eV	−1.50 eV	2.63 eV
CsPbI_1.4_Br_1.6_	−301.11 eV	−305.11 eV	−1.50 eV	2.51 eV

### 2.3 Healing of Br Vacancy Defects

**Figure 3:**
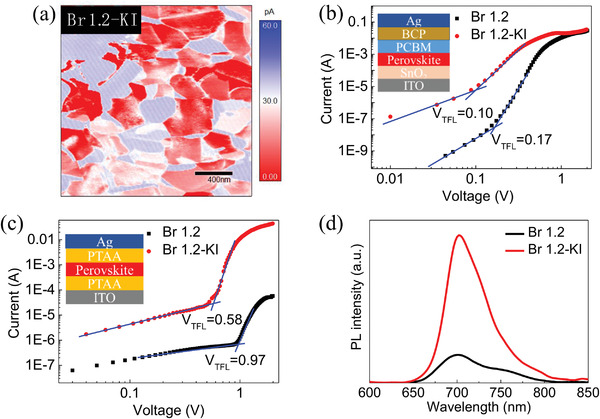
a) c‐AFM images of KI‐treated Br 1.2 film. Logarithm of *J*–*V* curves in the dark for Br 1.2 perovskite device without and with KI treatment, based on b) electron‐only structure and c) hole‐only structure (inset shows the device structure). d) PL spectra of perovskite film without and with KI treatment.

In this work, KI is added into the Br 1.2 precursor solution to heal the V_Br_. The I^−^ in KI is expected to compensate for vacancy defects and the generation of 2D K_2_PbX_4_ that distributed at the crystal surface is hopeful preventing Br escape from film surface.^[^ ^13^, ^33^ ^]^ To identify the optimum concentration of KI in the precursor solution, *J–V* characterizations of the KI‐treated devices were systematically studied (Figure S14, Supporting Information). The KI concentration of 5 mol% is proved as the optimal condition. As expected, the conductivity of the KI‐treated perovskite film drops rapidly due to the compensation of V_Br_ defects (**Figure** **3**a). Electron‐only and hole‐only devices were fabricated to calculate the trap‐state densities (*N*_trap_) (Figure 3b,c).^[^ ^34^ ^]^ The corresponding electron trap density is 3.25 × 10^15^ cm^−3^ for the reference film and 1.91 × 10^15^ cm^−3^ for the KI‐treated film. The hole‐trap density is 1.85 × 10^16^ cm^−3^ for the reference film and 1.11 × 10^16^ cm^−3^ for the KI‐treated film. The trap densities are all reduced, indicating that the KI addition contributes to a higher film quality. The hole trap‐state density is much higher than that of electron trap‐states in both reference and KI‐treated devices, indicating a buildup of positive charges in perovskite films and thus confirming the high concentration of V_Br_ in Br‐rich films. As shown in Figure 3d, the splitting of PL peak for Br 1.2 film still exists, which further demonstrates that the significantly reduced defect states mainly originates from the compensation of V_Br_ defects.

Figure S15a–d shows the surface scanning electron microscopy (SEM) images of the films treated with different KI concentrations. The typical grain size increases from 200 to 500 nm when the KI concentration is increased to 10 mol%. The SEM images of corresponding unannealed perovskite films prove that the differences in crystallization already occur during nucleation (Figure S16, Supporting Information), which can be explained by the decreased activation energy for crystallization.^[^ ^35^ ^]^ The larger grains provide a contracted specific surface for Br to escape, which is a considerable contribution to the decline of the charge defect density. Besides, the interpenetrating grains facilitate charge transport (Figure S15e,f, Supporting Information).

**Figure 4:**
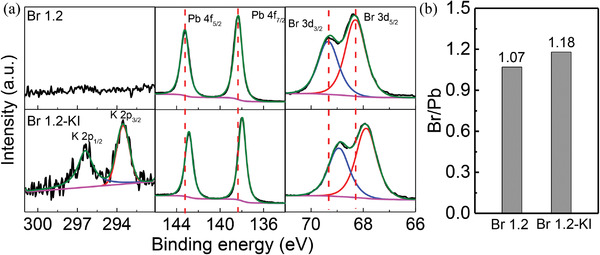
a) XPS of perovskite thin films with different Br contents. b) Fitted results of the Br/Pb ratio.

X‐ray photoelectron spectroscopy (XPS) was performed to gain further insight into the interaction between KI and PbX_2_, where X is a halide atom. As shown in **Figure** **4**a, the Pb 4f and Br 3d peaks of the KI‐treated film shift toward lower binding energy, confirming the intensive coordination effect between KI and PbBr_2_, which helps to anchor Br in the perovskite lattice.^[^ ^36^ ^]^ We conclude that the decrease of V_Br_ can be attributed to the immobilization of the halide atom through complexing with K, which prevents Br from escaping. The ratio of Br/Pb is used to monitor the relative Br content indirectly. As shown in Figure 4b, a higher Br/Pb ratio (1.18) is observed in the KI‐treated sample compared with the reference (1.07), which further confirms the Br‐vacancy lowering effect of KI.^[^ ^37^ ^]^ Figure S17 in the Supporting Information displays contact potential difference (CPD) distribution graphs under dark and illuminated conditions for the reference and the KI‐treated samples, respectively. The surface photovoltage difference (CPD_dark_−CPD_light_) for the KI‐treated sample (69 mV) is higher than that of the reference sample (46 mV). It suggests that more photo‐excited electrons are accumulated at the surfaces of the KI‐treated sample, inducing a greater shift in the electron quasi‐Fermi level toward the conduction band, due to the reduced nonradiative recombination in the KI‐treated sample.^[^ ^38^ ^]^

**Figure 5:**
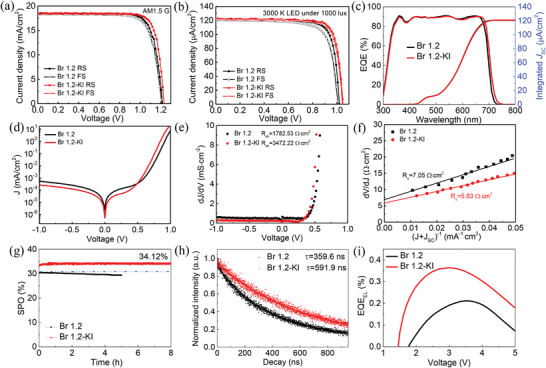
Typical *J*–*V* curves for Br 1.2 photovoltaic cells without and with KI treatment under a) AM1.5 G and b) 3000 K LED illumination. c) EQE curves of corresponding cells. Integrated *J*_SC_ can be calculated by integrating the EQE curve over the photon flux spectrum under 3000 K LED illumination. d) Dark *J–V* curves of Br 1.2 photovoltaic cells without and with KI treatment. e) *dJ*/*dV* with the fit used to determine *R*_sh_. f) *dV*/*dJ* with the fit used to determine *R*_s_. g) SPO measurement as a function of time under 3000 K LED illumination. h) TRPL spectra of the perovskite films without and with KI treatment. i) EQE_EL_ as a function of voltage.

**Figure** **5**a shows the *J*–*V* curves of the best‐performing Br 1.2 perovskite cells with and without KI additive under AM1.5 G illumination. With the addition of 5 mol% KI to the perovskite precursor solution, the device efficiency improved from 17.83% to 18.54% due to a simultaneous enhancement in *V*_OC_ and FF. As can be seen in Figure S18 in the Supporting Information, the present *V*_OC_ and PCE values are the highest values among recently reported perovskite solar cells with similar band gap. Furthermore, we measured the *J*–*V* characteristics of perovskite IPVs under 1000 lux LED illumination, the results are displayed in Figure 5b and **Table** **2**. The reference device shows a *V*_OC_ of 1.02 V, a *J*_SC_ of 121.1 µA cm^−2^, an FF of 79.7%, and a PCE of 32.61%. In comparison, the 5 mol% KI‐treated device reached a higher PCE of 34.60%, with *V*_OC_ = 1.05 V, *J*_SC_ = 121.9 µA cm^−2^, and FF = 81.6%. As shown in Figure S19 in the Supporting Information, the *V*_OC_ of 1.05 V is among the highest *V*_OC_ values reported so far, and the *V*_OC_ deficit (*E*_loss_) (*E*_loss_ = *E*_g_ − *qV*_OC_,) of 710 mV is one of the smallest values.

**Table 2:** Photovoltaic parameters of Br 1.2 perovskite cells without and with KI treatment, measured under standard AM1.5 G (10^5^ µW cm^−2^) and 3000 K (1000 lux) LED illumination

Measure condition	Device type	Scan direction	*J*_SC_ ^a)^	*V*_OC_ [V]	FF [%]	*P*_max_ [µW cm^−2^]	PCE [%]
AM1.5 G	Br 1.2	RS	18.39	1.22	79.4		17.83
		FS	18.37	1.21	77.9		17.27
	Br 1.2‐KI	RS	18.49	1.24	81.2		18.54
		FS	18.37	1.24	80.8		18.33
3000 K LED 1000 lux	Br 1.2	RS	121.1	1.02	79.7	98.45	32.61
		FS	120.6	1.01	75.6	92.09	30.50
	Br 1.2‐KI	RS	121.9	1.05	81.6	104.44	34.60
		FS	121.3	1.05	79.9	101.76	33.71

^a)^ The unit of *J*_SC_ in AM1.5 G is mA cm^−2^, the unit of *J*_SC_ in LED is µA cm^−2^.

The absorption edge of the perovskite material is red‐shifted due to the addition of iodide ions (Figure S20, Supporting Information), but the integrated *J*_SC_ shows negligible increase (Figure 5c), due to the small number of photons in this part of the LED spectrum. The improvement of efficiency is attributed to the enhancement of *V*_OC_ and FF, owing to the healing of V_Br_ defects. Our results indicate that besides halide segregation, the V_Br_ defects in the perovskite absorbers is one of the dominant factors responsible for the *V*_OC_ deficit in Br‐rich wide‐bandgap perovskite IPVs.

The FF of photovoltaic cells is determined by *R*_s_ and *R*_sh_ of the device. Since the *R*_s_ and *R*_sh_ play competitive dominant roles under different light intensities, the FF of devices shows a maximum value at a certain light intensity.^[^ ^39^ ^]^ Generally, the smaller the light intensity corresponding to the maximum value, the fewer the trap that causes recombination inside the perovskite film.^[^ ^40^, ^41^ ^]^ The maximum FF of Br 1.2‐KI device occurs at 600 lux, which is far smaller than the Br 1.2 counterpart, indicating that the defects in Br 1.2‐KI thin film have been passivated (Figures S20 and S21 and Table S2, Supporting Information). As can be seen in the dark *J–V* characterization of IPVs (Figure 5d), the leakage currents are significantly reduced with the introduction of KI. The enlarged *R*_sh_ from 1782.53 to 3472.22 Ω cm^2^ and reduced *R*_s_ from 7.05 to 5.83 Ω cm^2^ contribute the improvement of FF that from 75.6% to 79.9% (Figure 5e,f).^[^ ^42^ ^]^ Additionally, the *V*_OC_ is increased from 1.01 to 1.05 V for the IPVs and from 1.21 to 1.24 V for the solar cells.

Subsequently, the stabilized‐power‐output (SPO) of the unencapsulated devices was measured in ambient air at an RH of ± 60% and at room temperature (25 °C) to evaluate their operating performance. The KI‐treated cell was monitored while applying 0.91 V forward bias under continuous indoor illumination (3000 K LED, 1000 lux). The cell exhibits a stable SPO of 34.12% within 8 h (Figure 5g). This is consistent with the PCE obtained from the *J*–*V* curve in Figure 5b. Thus, the KI‐treated device shows outstanding operational stability, while in comparison, the reference device shows a declining efficiency starting at 30.40%, and gradually decreasing further to 29.37% over 5 h of illumination. Likewise, under continuous simulated sunlight (AM1.5 G, 100 mW/cm^2^), the reference device displays a fast decline, and the KI‐treated device maintains their initial PCEs during 1000 s of illumination (Figure S24, Supporting Information).

To further evaluate the effect of the introduction of KI on the recombination dynamics of the devices, we compared the corresponding charge‐recombination lifetime using transient photovoltage measurements. The KI‐treated device exhibits a higher photovoltage and longer charge‐recombination lifetime (2.09 µs) than its counterpart (1.56 µs) (Figure S25, Supporting Information). The prolonged recombination lifetime is associated with the suppressed charge recombination at the interface. The time‐resolved photoluminescence (TRPL) results (Figure 5h) reveal that the KI‐treated perovskite film exhibits longer mean carrier lifetime (591.9 ns) than the reference sample (391.6 ns). Here, the TRPL conforms to a single exponential decay process, which was fitted by single exponential equation *y* = *A*_0_+*A*_1_exp(−*x*/*t*_1_). Fundamentally, the nonradiative recombination losses can be quantified by measuring the external electroluminescence quantum efficiency (EQE_EL_). We tested the photovoltaic device operating as an LED in the dark and under a voltage bias. Due to the severe nonradiative recombination losses (> 240 mV) in Br‐rich wide‐bandgap cells, it is almost impossible to accurately calculate the nonradiative compound *V*_OC_ using the reported calculation methods.^[^ ^43^, ^44^ ^]^ It is encouraging that the EQE_EL_ for KI‐treated cell reaches 0.36% for higher driving currents, placing the solar cell among the most efficient perovskite LEDs as well, emitting in the near‐infrared/red spectral range, while the EQE_EL_ for the reference sample reaches 0.21% and emits at weaker light intensity (Figure 5i). These values for our photovoltaic devices indicate that the origin of nonradiative recombination is reduced by strongly suppressing vacancy defects.

### 2.4 High Efficiency Perovskite Indoor Photovoltaic Modules

**Figure 6:**
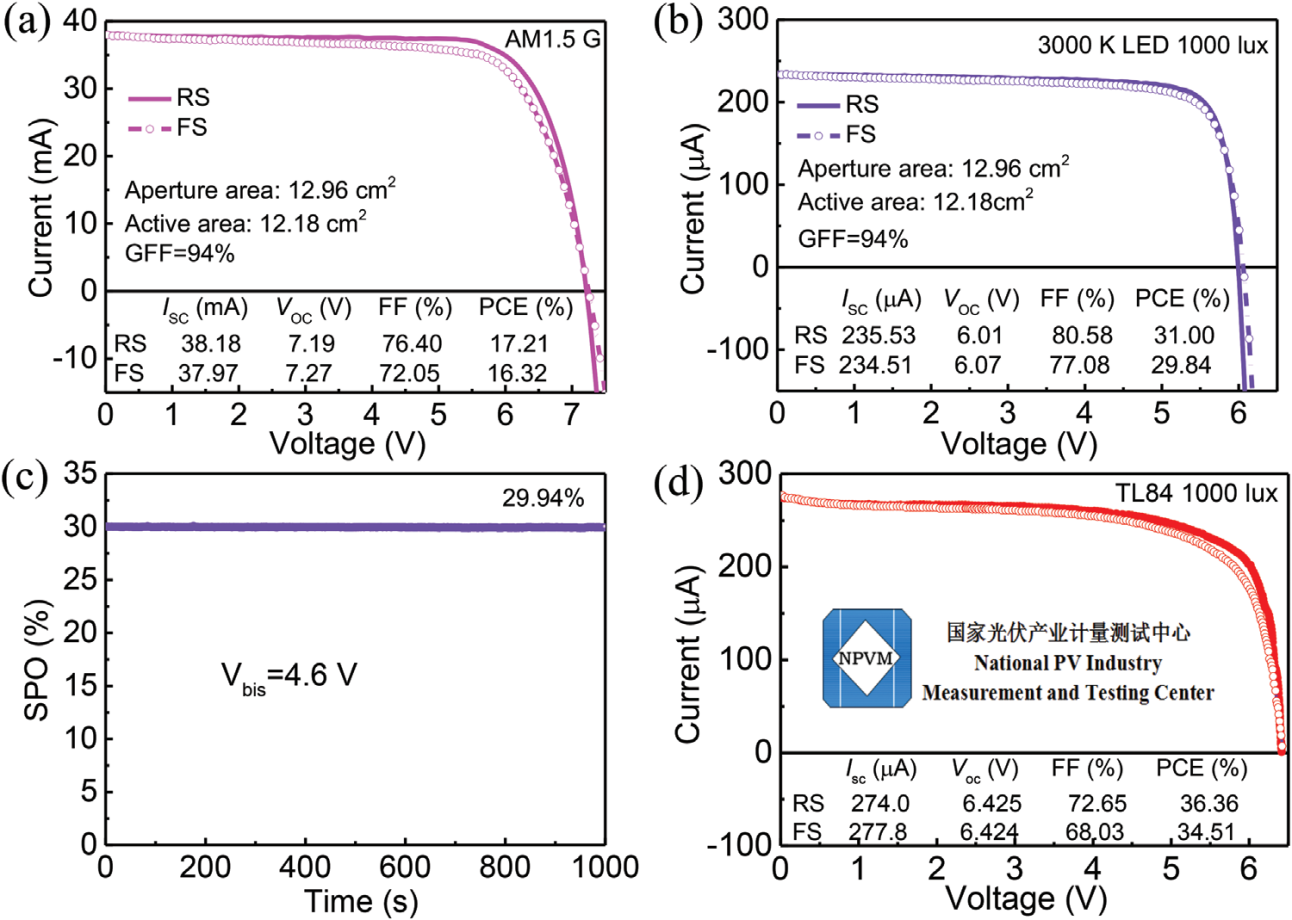
a) The *J*–*V* characteristics measured under AM1.5 G of the best‐performing KI‐treated Br 1.2 perovskite module. b) The *J*–*V* characteristics measured under 3000 K LED of the best‐performing KI‐treated Br 1.2 perovskite module. c) The SPO measurement of the module held at 4.6 V forward bias as a function of time. d) Certificated *J*–*V* curves of our perovskite indoor photovoltaic module under TL84 light (1000 lux) according to IEC60904 and SEMI PV89 test standards, measured at Chinese National Photovoltaic Industry Measurement and Testing Center (NPVM).

The perovskite IPVs offer promising industrialization opportunities as its efficiency surpasses that all other photovoltaic cells and the mild indoor environment facilitates a long lifetime. Fabricating modules with subcells connected in series is needed to obtain high voltage to drive the electronics working. Subsequently, we fabricated six sub‐cells based perovskite indoor photovoltaic modules with aperture area of 12.96 cm^2^ and geometric FF of 94% (Figure S26, Supporting Information). The module shows a high aperture/active area efficiency of 16.18%/17.21% under standard AM1.5 G simulated sunlight (**Figure** **6**a), leading to a small cell‐to‐module (CTM) efficiency loss.^[^ ^45^, ^46^ ^]^ The home‐test aperture area efficiency of the perovskite module under 1000 lux LED illumination is 29.14%, corresponding to an active area efficiency of 31.00% (*I*_SC_ = 235.53 µA, *V*_OC_ = 6.01 V, and FF = 80.58%), which is far superior than previous reports (Figure 6b). The small CTM efficiency loss is particularly noteworthy for an indoor weak light photovoltaic module, as they are extremely sensitive to leakage current. The corresponding SPO performance was recorded at a forward bias of 4.6 V (near the maximum power point) and a stabilized PCE of 29.94% was determined after 1000 s (Figure 6c). We sent our modules to Chinese National Photovoltaic Industry Measurement and Testing Center (NPVM) for efficiency certification based on IEC60904 and SEMI PV89 test standards by using the third generation of low light solar cell simulator and perovskite solar cell performance measurement system. They offered a comprehensive test by using multiple light sources including U30, CWF, D65, and TL84, with the spectra shown in Figure S27 in the Supporting Information. The corresponding active area efficiencies of the best‐performing perovskite indoor photovoltaic modules are, respectively, 35.67%, 34.37%, 34.71%, and 36.36% (Figure 6d and Figure S28, Supporting Information). This is the first report on indoor photovoltaic certification of perovskite module, which is of great significance for promoting the standard and sustainable development of this field (Table S3, Supporting Information). Interestingly, an indoor light energy harvesting system, consisting of mainboard, perovskite indoor photovoltaic module, temperature and humidity sensors, supercapacitor, Bluetooth, and mobile App was designed in this work (Figure S29, Supporting Information). The system enables indoor light photovoltaic conversion, energy storage, temperature and humidity sensing, and communication with smartphones.

## 3 Conclusion

In summary, we fabricated perovskite photovoltaic cells with varying I/Br ratios and optimized them, taking into account the limit parameters of IPVs according to the S‐Q limit formula. We found that perovskite materials with an ideal band gap generally contain a high Br content, while DFT and c‐AFM show that this high Br content leads to a high V_Br_ defect density, resulting in aggravating nonradiative recombination and *V*_OC_ deficit. Subsequently, KI treatment is introduced to heal the V_Br_ defects, thus a homogenous and stable perovskite film is formed. As a result, the *V*_OC_ is increased under low light illumination, contributing to a certified efficiency of 36.36% for a perovskite indoor photovoltaic module. Our results reveal that, the *V*_OC_ deficit is also dominated by the V_Br_ apart from halide segregation. The module is capable to power an indoor light energy harvesting system under 507 lux LED illumination that monitors temperature and humidity through a sensor and displays the result on a smartphone via Bluetooth. We believe that this work provides significant theoretical and technical support for commercial indoor applications of perovskite technology.

## 4 Experimental Section

### Materials and Methods

Lead iodide (PbI_2_, >99.99%), lead bromide (PbBr_2_, >99.99%), cesium iodide (CsI, ≥99.99%), poly[bis(4‐phenyl) (2,4,6‐trimethylphenyl) amine] (PTAA, *M*_n_ = 6000–15 000), formamidinium iodide (FAI, ≥99.5%), 2,9‐dimethyl‐4,7‐diphenyl‐1,10‐phenanthroline (BCP, >99%), and phenethylammonium iodide (PEAI, ≥99.5%) were purchased from Xi'an polymer Light Technology Corp. [6,6]‐phenyl‐C61‐butyric acid methyl ester (PCBM) was purchased from Lumtec. Potassium iodide (KI, ≥99.99%), dimethyl sulfoxide (DMSO, ≥99.9%), *N*,*N*‐dimethylformamide (DMF, 99.8%), chlorobenzene (99.8%), isopropanol (IPA, ≥99.9%), and ethyl acetate (99%) were purchased from Aladdin.  Aluminum oxide nanoparticles (Al_2_O_3_, <50 nm particle size, 20 wt% in isopropanol) were purchased from Sigma‐Aldrich.

### Fabrication of Photovoltaic Cell

ITO‐coated glass (OPV Tech Co., Ltd.) substrate with sheet resistance of 17 Ω sq^−1^ was sequentially washed by ultrasonication with deionized water and ethanol for 10 min and then treated with oxygen plasma for 10 min. Then, a precursor of PTAA in chlorobenzene (1.5 mg mL^−1^) was spin‐coated onto ITO substrate at 4000 rpm for 30 s, followed by annealing at 120 °C for 15 min. Afterward, Al_2_O_3_ nanoparticles ink with diluted concentration (1:50 V:V diluted in isopropanol) was spin‐coated at 3500 rpm for 30 s, and then annealed at 120 °C for 15 min. The perovskite thin film (Cs_0.17_FA_0.83_PbI_3−_ *_x_* Br*_x_*) was deposited by using one‐step spin‐coating method. The perovskite precursor solution (1.2 M) was comprised by mixing CsI, FAI, PbI_2_, and PbBr_2_ in DMF:DMSO (7:3, v:v) and stirred for 6 h. KI (5 mol%) and PbI_2_ (5 mol%) were both added to form the KI‐doped perovskite precursor solution. The perovskite solution was spin coated with two‐step program that 2000 rpm for 3 s and 4000 rpm for 30 s, respectively. During the second step, 300 µL of ethyl acetate was dropped on spinning substrate 10 s prior to end of the program. Then, the film was annealed at 120 °C for 20 min. The formation of PEAI‐based 2D perovskite and PCBM layers was followed our previous report.^[^ ^26^ ^]^ Subsequently, the interfacial layer BCP was deposited by spin‐coating a saturated solution in isopropanol at 4000 rpm for 30 s. Finally, the Ag electrode (120 nm) was evaporated on BCP layer. Note that all the solutions were filtered with 0.22 µm polyvinylidene fluoride (PVDF) filters before spin coating and all the spin processes were performed in the dry air‐filled glovebox.

### Fabrication of Photovoltaic Module

The ITO glass was scribed using a nanosecond laser machine with power of 12.5 W and scan speed of 1200 mm s^−1^ to form P1 lines. The fabrication process of modules was the same as the photovoltaic cell. After the deposition of the BCP film, the sample was re‐etched to form P2 lines. Here the power was 0.25 W and scan speed was 680 mm s^−1^. Finally, it formed effective monolithically interconnected modules by scribing the Ag electrode using 0.25 W power and 800 mm s^−1^ scan speed to form P3 lines.

### Characterizations

The crystal structure was characterized by Bruker D8 Advance X‐ray diffractometer with Cu K*α* radiation at 40 kV and 40 mA. The surface and cross‐sectional morphology of thin films were analyzed by using SEM (FEI ApreoLoVac). The *J*–*V* curves were characterized by using a digital source meter (Keithley 2400) and a Newport solar simulator (ORIEL‐SOI3A) with AM1.5 G spectrum. The light intensity was adjusted to 1000 W m^−2^ using a standard Si cell (91 150 V). A black mask with an aperture area of 0.09 cm^2^ was placed on top of the device to control the illuminated area. For indoor light conditions, SGX T5 5 W LED (3000 K) was applied as light source, and the illumination was calibrated to 1000 lux by illuminometer (TES‐1330A). Spectrum of the indoor light source (SGX T5 5 W LED 3000 K, SGX T5 5 W LED 6000 K, FSL T5 8 W FL 2700 K, FSL T5 8 W FL 6500 K) was collected using a spectral illuminometer (SPIC‐200, Everfine). The indoor light power density was obtained based on light spectrum and luminosity function (Note S2, Supporting Information). The EQE of perovskite photovoltaic cells were measured by using spectra response system (Enlitech QE‐R), with Si solar cell as a reference. The PL and TRPL spectra were recorded by using an FV1200 laser scanning confocal microscopy in which a 532 nm of pulsed diode laser was used for excitation with repetition rate of 40 MHz and the emission was filtered through 50/50 dichroic beam splitter as well as 700–800 nm long pass filter. The EL spectrum of the perovskite LED was recorded simultaneously by a commercialized system (XPQY‐EQE‐350‐1100, Guangzhou Xi Pu Optoelectronics Technology Co., Ltd.) that is equipped with an integrated sphere (GPS‐4P‐SL, Labsphere) and a photodetector array (S7031‐1006, Hamamatsu Photonics). The XPS characterizations were obtained with a Thermo Fisher Scientific K‐ALPHA^+^, using the HeI (21.22 eV) emission line and Al K*α* radiation (1486.6 eV). ORCA module on the Asylum Research Cypher ES was used for c‐AFM measurement. The c‐AFM measurement was held under 15 µW cm^−2^ illumination.

### First‐Principles Calculations

The DFT calculations were carried out using the Vienna Ab‐initio Simulation Package (VASP) with the frozen‐core all‐electron projector‐augment‐wave method.^[^ ^47^, ^48^, ^49^, ^50^ ^]^ The Perdew–Burke–Ernzerhof of generalized gradient approximation was adopted to describe the exchange and correlation potential.^[^ ^51^ ^]^ The cut‐off energy for the plane‐wave basis set was set to 450 eV. The supercell of CsPbI_2.4_Br_0.6_, CsPbI_1.8_Br_1.2_, and CsPbI_1.4_Br_1.6_ used in this study was 2 × 2 × 5. The geometry optimizations were performed until the forces on each ion was reduced below 0.01 eV Å^−1^, and the gamma k‐point sampling of the Brillouin zone was used for all calculations.^[^ ^52^ ^]^ The formation energies of Br‐vacancy were calculated by the following formulas

(1) $$E_{\mathrm{form}}=E_{\left(\mathrm{vacancy}\right)}-E_{\left(\mathrm{supercell}\right)}+\mu_{\left(\mathrm{atom}\right)}$$

where *E*_(vacancy)_ and *E*_(supercell)_ are the total energies of CsPbI_2.4_Br_0.6_, CsPbI_1.8_Br_1.2_ and CsPbI_1.4_Br_1.6_ with and without vacancy, respectively. The *µ*_(atom)_ is the chemical potentials of Br atom.

## Conflict of Interest

The authors declare no conflict of interest.

## Supporting information

Supporting Information

## Data Availability

The data that support the findings of this study are available from the corresponding author upon reasonable request.

## References

[1] Y. Cui , H. Yao , T. Zhang , L. Hong , B. Gao , K. Xian , J. Qin , J. Hou , Adv. Mater. 2019, 31, 1904512. 10.1002/adma.201904512 31490601

[2] Y. Li , Z. Chi , X. Liu , T. Zhu , in Proc. 16th ACM Conf. Embedded Networked Sensor Systems (Ed: G. S. Ramachandran ), ssociation for Computing Machinery, New York, 2018, pp. 159–171.

[3] W. Shockley , H. J. Queisser , J. Appl. Phys. 1961, 32, 510.

[4] A. Venkateswararao , J. K. Ho , S. K. So , S.‐W. Liu , K.‐T. Wong , Mater. Sci. Eng., R 2020, 139, 100517.

[5] E. Saloux , A. Teyssedou , M. Sorin , Sol. Energy 2011, 85, 713.

[6] O. Y. Gong , G. S. Han , S. Lee , M. K. Seo , C. Sohn , G. W. Yoon , J. Jang , J. M. Lee , J. H. Choi , D.‐K. Lee , ACS Energy Lett. 2022, 7, 2893.

[7] Y. Cui , L. Hong , T. Zhang , H. F. Meng , H. Yan , F. Gao , J. H. Hou , Joule 2021, 5, 1016.

[8] X. Wang , Y. Ling , X. Lian , Y. Xin , K. B. Dhungana , F. Perez‐Orive , J. Knox , Z. Chen , Y. Zhou , D. Beery , K. Hanson , J. Shi , S. Lin , H. Gao , Nat. Commun. 2019, 10, 695. 30741944 10.1038/s41467-019-08610-6 PMC6370784

[9] M. Suri , A. Hazarika , B. W. Larson , Q. Zhao , M. Vallés‐Pelarda , T. D. Siegler , M. K. Abney , A. J. Ferguson , B. A. Korgel , J. M. Luther , ACS Energy Lett. 2019, 4, 1954.

[10] H. Zhang , X. Fu , Y. Tang , H. Wang , C. Zhang , W. Y. William , X. Wang , Y. Zhang , M. Xiao , Nat. Commun. 2019, 10, 1088. 30842434 10.1038/s41467-019-09047-7 PMC6403211

[11] Z. Guo , A. K. Jena , I. Takei , M. Ikegami , A. Ishii , Y. Numata , N. Shibayama , T. Miyasaka , Adv. Funct. Mater. 2021, 31, 2103614.

[12] R. A. Belisle , K. A. Bush , L. Bertoluzzi , A. Gold‐Parker , M. F. Toney , M. D. McGehee , ACS Energy Lett. 2018, 3, 2694.

[13] Y. Zhao , P. Miao , J. Elia , H. Hu , X. Wang , T. Heumueller , Y. Hou , G. J. Matt , A. Osvet , Y.‐T. Chen , M. Tarrago , D. De Ligny , T. Przybilla , P. Denninger , J. Will , J. Zhang , X. Tang , N. Li , C. He , A. Pan , A. J. Meixner , E. Spiecker , D. Zhang , C. Brabec , Nat. Commun. 2020, 11, 6328. 33303755 10.1038/s41467-020-20066-7 PMC7730187

[14] G. Nan , X. Zhang , M. Abdi‐Jalebi , Z. Andaji‐Garmaroudi , S. D. Stranks , G. Lu , D. Beljonne , Adv. Energy Mater. 2018, 8, 1702754.

[15] A. Musiienko , P. Moravec , R. Grill , P. Praus , I. Vasylchenko , J. Pekarek , J. Tisdale , K. Ridzonova , E. Belas , L. Landová , Energy Environ. Sci. 2019, 12, 1413.

[16] A. Wang , Z. Cao , J. Wang , S. Wang , C. Li , N. Li , L. Xie , Y. Xiang , T. Li , X. Niu , J. Energy Chem. 2020, 48, 426.

[17] Y. Yang , J. Wu , X. Wang , Q. Guo , X. Liu , W. Sun , Y. Wei , Y. Huang , Z. Lan , M. Huang , Adv. Mater. 2020, 32, 1904347. 10.1002/adma.201904347 31880354

[18] J. Kang , L.‐W. Wang , J. Phys. Chem. Lett. 2017, 8, 489. 28071911 10.1021/acs.jpclett.6b02800

[19] Y. Cui , L. Hong , J. Hou , ACS Appl. Mater. Interfaces 2020, 12, 38815. 32805933 10.1021/acsami.0c10444

[20] M. J. Wu , C. C. Kuo , L. S. Jhuang , P. H. Chen , Y. F. Lai , F. C. Chen , Adv. Energy Mater. 2019, 9, 1901863.

[21] J. Liang , P. Zhao , C. Wang , Y. Wang , Y. Hu , G. Zhu , L. Ma , J. Liu , Z. Jin , J. Am. Chem. Soc. 2017, 139, 14009. 28933843 10.1021/jacs.7b07949

[22] E. C. Shen , J. D. Chen , Y. Tian , Y. X. Luo , Y. Shen , Q. Sun , T. Y. Jin , G. Z. Shi , Y. Q. Li , J. X. Tang , Adv. Sci. 2020, 7, 1901952. 10.1002/advs.201901952 PMC6947708 31921565

[23] C. Zhou , J. Zhang , Y. Zhu , Y. Liu , L. Li , J. Power Sources 2021, 494, 229726.

[24] D. Luo , W. Yang , Z. Wang , A. Sadhanala , Q. Hu , R. Su , R. Shivanna , G. F. Trindade , J. F. Watts , Z. Xu , Science 2018, 360, 1442. 29954975 10.1126/science.aap9282

[25] J. W. Lim , H. Kwon , S. H. Kim , Y.‐J. You , J. S. Goo , D.‐H. Ko , H. J. Lee , D. Kim , I. Chung , T. G. Kim , Nano Energy 2020, 75, 104984.

[26] C. Zhang , S. Wu , L. Tao , G. M. Arumugam , C. Liu , Z. Wang , S. Zhu , Y. Yang , J. Lin , X. Liu , Adv. Energy Mater. 2020, 10, 2002004.

[27] W. Q. Wu , P. N. Rudd , Z. Ni , C. H. Van Brackle , H. Wei , Q. Wang , B. R. Ecker , Y. Gao , J. S. Huang , J. Am. Chem. Soc. 2020, 142, 3989. 32031790 10.1021/jacs.9b13418

[28] S. Draguta , O. Sharia , S. J. Yoon , M. C. Brennan , Y. V. Morozov , J. S. Manser , P. V. Kamat , W. F. Schneider , M. Kuno , Nat. Commun. 2017, 8, 200. 28779144 10.1038/s41467-017-00284-2 PMC5544754

[29] G. Xia , B. Huang , Y. Zhang , X. Zhao , C. Wang , C. Jia , J. Zhao , W. Chen , J. Li , Adv. Mater. 2019, 31, 1902870. 10.1002/adma.201902870 31322309

[30] B. Huang , G. Kong , E. N. Esfahani , S. Chen , Q. Li , J. Yu , N. Xu , Y. Zhang , S. Xie , H. Wen , npj Quantum Mater. 2018, 3, 30.

[31] C. Chen , Z. Song , C. Xiao , R. A. Awni , C. Yao , N. Shrestha , C. Li , S. S. Bista , Y. Zhang , L. Chen , ACS Energy Lett. 2020, 5, 2560.

[32] Y. Yu , C. Wang , C. R. Grice , N. Shrestha , D. Zhao , W. Liao , L. Guan , R. A. Awni , W. Meng , A. J. Cimaroli , ACS Energy Lett. 2017, 2, 1177.

[33] L. Kuai , Y. Wang , Z. Zhang , Y. Yang , Y. Qin , T. Wu , Y. Li , Y. Li , T. Song , X. Gao , Sol. RRL 2019, 3, 1900053.

[34] C. Liu , J. Tu , X. Hu , Z. Huang , X. Meng , J. Yang , X. Duan , L. Tan , Z. Li , Y. Chen , Adv. Funct. Mater. 2019, 29, 1808059.

[35] T. Bu , X. Liu , Y. Zhou , J. Yi , X. Huang , L. Luo , J. Xiao , Z. Ku , Y. Peng , F. Huang , Energy Environ. Sci. 2017, 10, 2509.

[36] Y. Mo , C. Wang , X. Zheng , P. Zhou , J. Li , X. Yu , K. Yang , X. Deng , H. Park , F. Huang , J. Alloys Compd. 2022, 1, 309.

[37] L. G. Wang , H. P. Zhou , J. N. Hu , B. L. Huang , M. Z. Sun , B. W. Dong , G. H. J. Zheng , Y. Huang , Y. H. Chen , L. Li , Z. Q. Xu , N. X. Li , Z. Liu , Q. Chen , L. D. Sun , C. H. Yan , Science 2019, 363, 265. 30655439 10.1126/science.aau5701

[38] M. Lee , E. Choi , A. M. Soufiani , J. Lim , M. Kim , D. Chen , M. A. Green , J. Seidel , S. Lim , J. Kim , Adv. Funct. Mater. 2021, 31, 2008908.

[39] H. Kanda , V. D. Mihailetchi , M. E. Gueunier‐Farret , J. P. Kleider , Z. Djebbour , J. Alvarez , B. Philippe , O. Isabella , M. R. Vogt , R. Santbergen , J. Alloys Compd. 2022, 1, 148.

[40] M. A. Green , Solid‐State Electron. 1981, 24, 788.

[41] R. Steim , T. Ameri , P. Schilinsky , C. Waldauf , G. Dennler , M. Scharber , C. J. Brabec , Sol. Energy Mater. Sol. Cells 2011, 95, 3256.

[42] S. S. Hegedus , W. N. Shafarman , Prog. Photovoltaics 2004, 12, 155.

[43] M. Saliba , T. Matsui , K. Domanski , J.‐Y. Seo , A. Ummadisingu , S. M. Zakeeruddin , J.‐P. Correa‐Baena , W. R. Tress , A. Abate , A. Hagfeldt , Science 2016, 354, 206. 27708053 10.1126/science.aah5557

[44] Q. Jiang , Y. Zhao , X. W. Zhang , X. L. Yang , Y. Chen , Z. M. Chu , Q. F. Ye , X. X. Li , Z. G. Yin , J. B. You , Nat. Photonics 2019, 13, 460.

[45] S. S. Chen , X. Z. Dai , S. Xu , H. Y. Jiao , L. Zhao , J. S. Huang , Science 2021, 373, 902. 34413234 10.1126/science.abi6323

[46] Y. H. Deng , S. Xu , S. S. Chen , X. Xiao , J. J. Zhao , J. S. Huang , Nat. Energy 2021, 6, 633.

[47] G. Kresse , J. Furthmüller , Phys. Rev. B 1996, 54, 11169. 10.1103/physrevb.54.11169 9984901

[48] G. Kresse , J. Furthmüller , J. Hafner , Phys. Rev. B 1994, 49, 14251. 10.1103/physrevb.49.14251 10010505

[49] G. Kresse , D. Joubert , Phys. Rev. B 1999, 59, 1758.

[50] P. E. Blöchl , Phys. Rev. B 1994, 50, 17953. 10.1103/physrevb.50.17953 9976227

[51] B. Hammer , L. B. Hansen , J. K. Nørskov , Phys. Rev. B 1999, 59, 7413.

[52] H. J. Monkhorst , J. D. Pack , Phys. Rev. B 1976, 13, 5188.

