# Supplementary material for: Br Vacancy Defects Healed Perovskite Indoor Photovoltaic Modules with Certified Power Conversion Efficiency Exceeding 36%
Source: Adv Sci (Weinh). 2022 Oct 17;9(33):2204138. doi: 10.1002/advs.202204138 (PMC9685472; doi:10.1002/advs.202204138)
Supplement: Supplementary file 1 — Supporting Information [file ADVS-9-2204138-s001.pdf]

ADVANCED SCIENCE
Open Access

Supporting Information

for *Adv. Sci.*, DOI 10.1002/advs.202204138

Br Vacancy Defects Healed Perovskite Indoor Photovoltaic Modules with Certified Power Conversion Efficiency Exceeding 36%

Cuiling Zhang, Chong Liu, Yanyan Gao, Shusheng Zhu, Fang Chen, Boyuan Huang, Yi Xie, Yaqing Liu, Mengen Ma, Zhen Wang, Shaohang Wu, Ruud E. I. Schropp and Yaohua Mai**

Supporting Information

Br Vacancy Defects Healed Perovskite Indoor Photovoltaic Modules with Certified Power Conversion Efficiency Exceeding 36%

Cuiling Zhang, Chong Liu, Yanyan Gao, Shusheng Zhu, Fang Chen, Boyuan Huang, Yi Xie, Yaqing Liu, Mengen Ma, Zhen Wang, Shaohang Wu, * *Ruud E. I. Schropp, Yaohua Mai* *

C. Zhang, Dr. C. Liu, Y. Gao, S. Zhu, Y. Xie, Y. Liu, M. Ma, Dr. S. Wu, Prof. R.E.I. Schropp, Prof. Y. Mai
Institute of New Energy Technology
College of Information Science and Technology, and Guangdong Engineering Research Center of Thin-Film Photovoltaic Processes and Equipment
Jinan University
Guangzhou 510632, China
E-mail: wushaohang@jnu.edu.cn, yaohuamai@jnu.edu.cn
Dr. F. Chen, Dr. B. Huang
Department of Materials Science and Engineering
Southern University of Science and Technology
Shenzhen 518055, Guangdong, China
Dr. Z. Wang,
Institute for Advanced Materials and Guangdong Provincial Key Laboratory of Optical Information Materials and Technology, South China Academy of Advanced Optoelectronics
South China Normal University
Guangzhou 510006, China

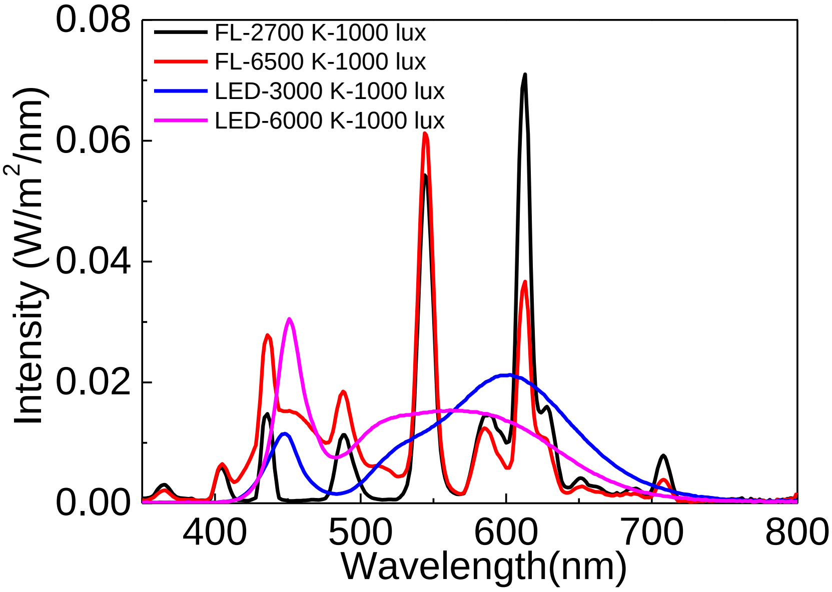

Figure S1. The emission spectra of FL (2700 K and 6500 K, 1000 lux) and LED (3000 K and 6000 K, 1000 lux) light source.

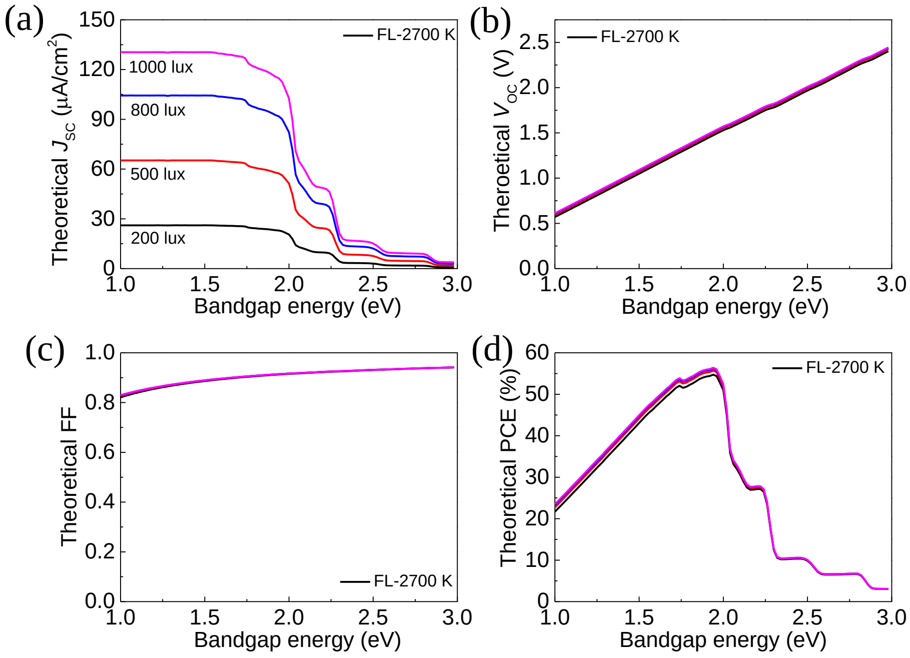

Figure S2. The maximum theoretical photovoltaic parameters of a single-junction solar cell as calculated by the S-Q limit model as a function of bandgap energy. The incident light spectra are taken from the 2700 K FL light sources at different illuminances (200, 500, 800 and 1000 lux).

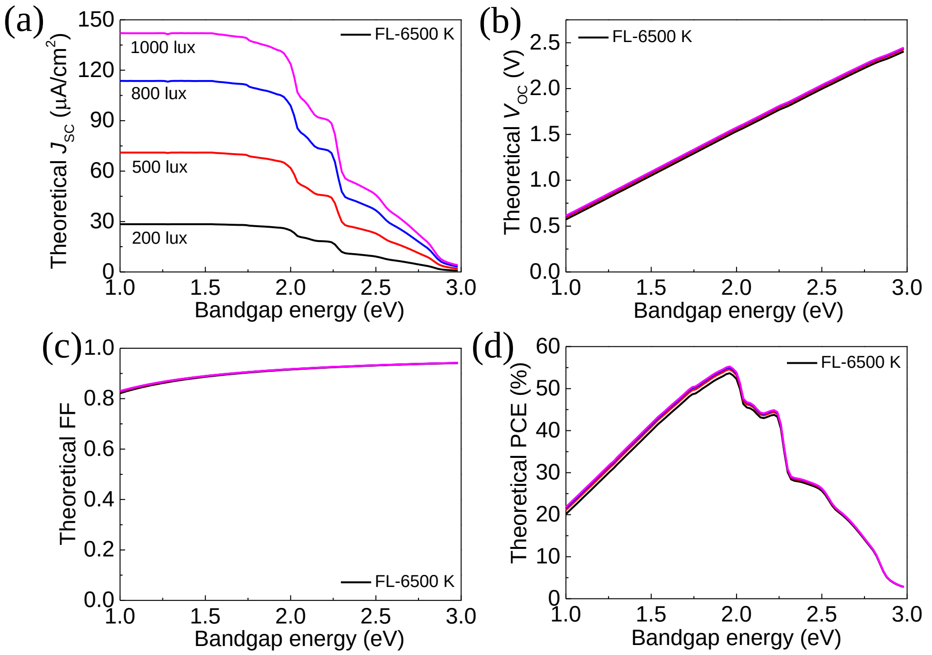

Figure S3. The maximum theoretical photovoltaic parameters of a single-junction solar cell as calculated by the S-Q limit model as a function of bandgap energy. The incident light spectra are taken from the 6500 K FL light sources at different illuminances (200, 500, 800 and 1000 lux).

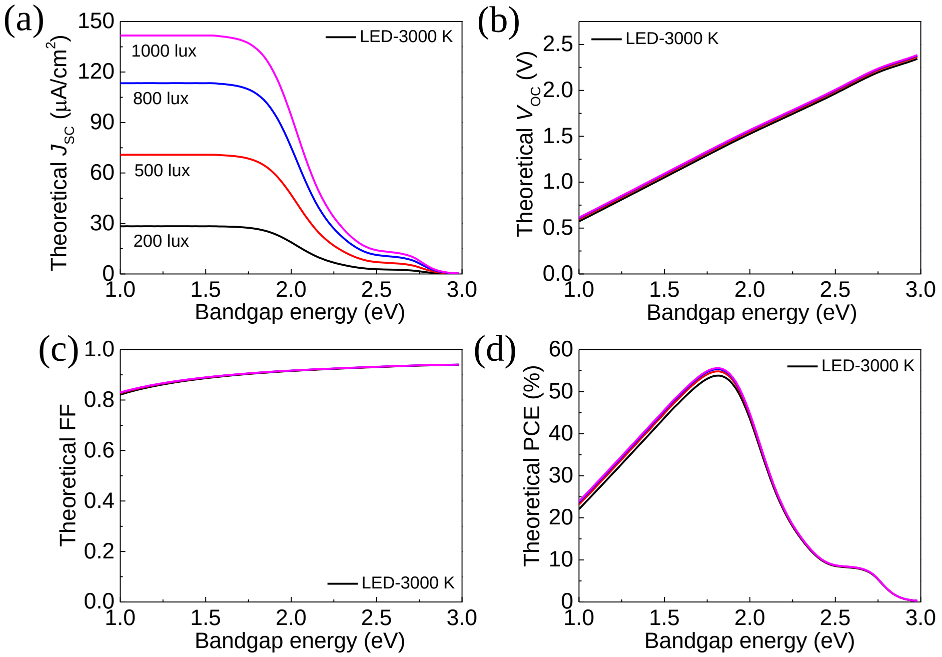

Figure S4. The maximum theoretical photovoltaic parameters of a single-junction solar cell as calculated by the S-Q limit model as a function of bandgap energy. The incident light spectra are taken from the 3000 K LED light sources at different illuminances (200, 500, 800 and 1000 lux).

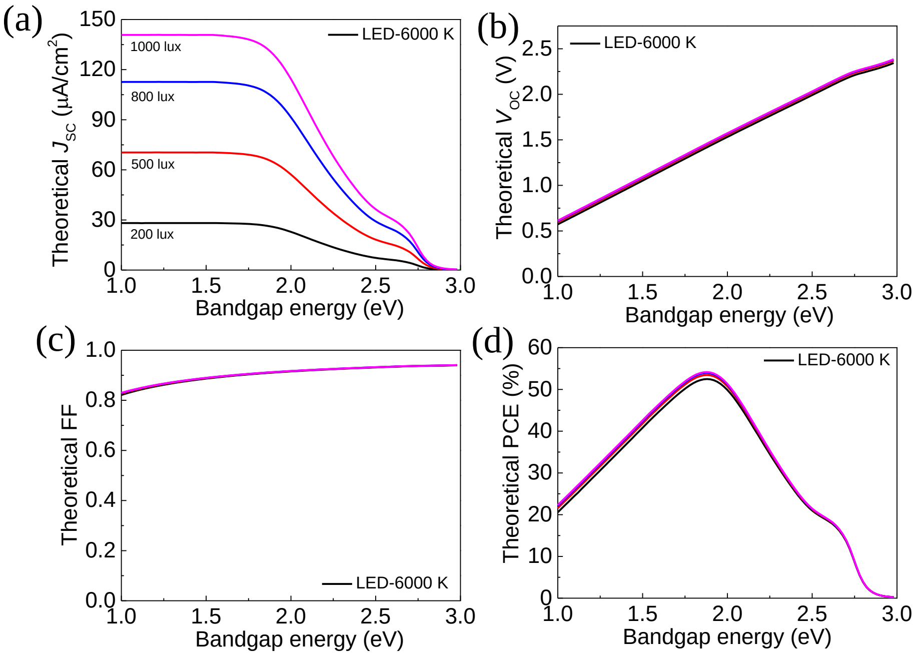

Figure S5. The maximum theoretical photovoltaic parameters of a single-junction solar cell as calculated by the S-Q limit model as a function of bandgap energy. The incident light spectra are taken from the 6000K LED light sources at different illuminances (200, 500, 800 and 1000 lux).

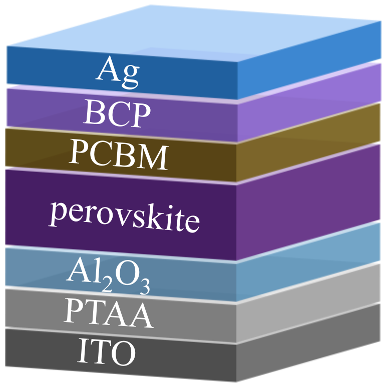

Figure S6. Schematic view of the structure of perovskite photovoltaic cell.

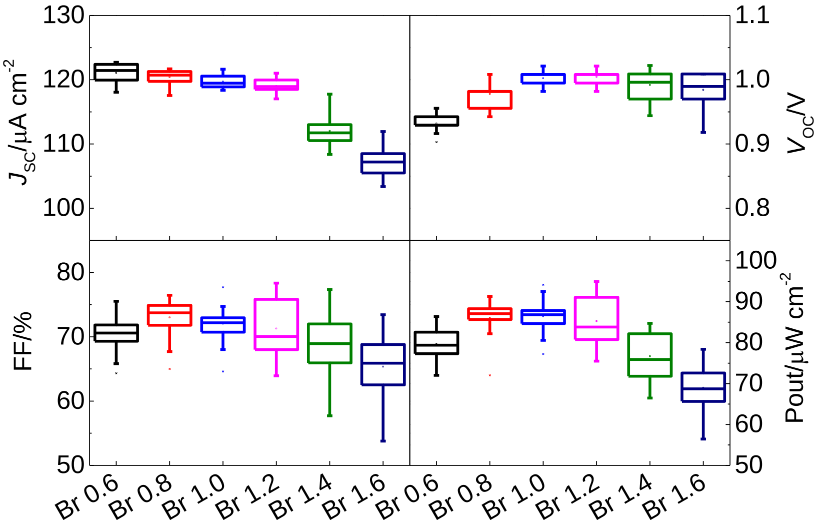

Figure S7. The statistics of parameters change of perovskite IPVs (devices number = 50) with varying Br contents.

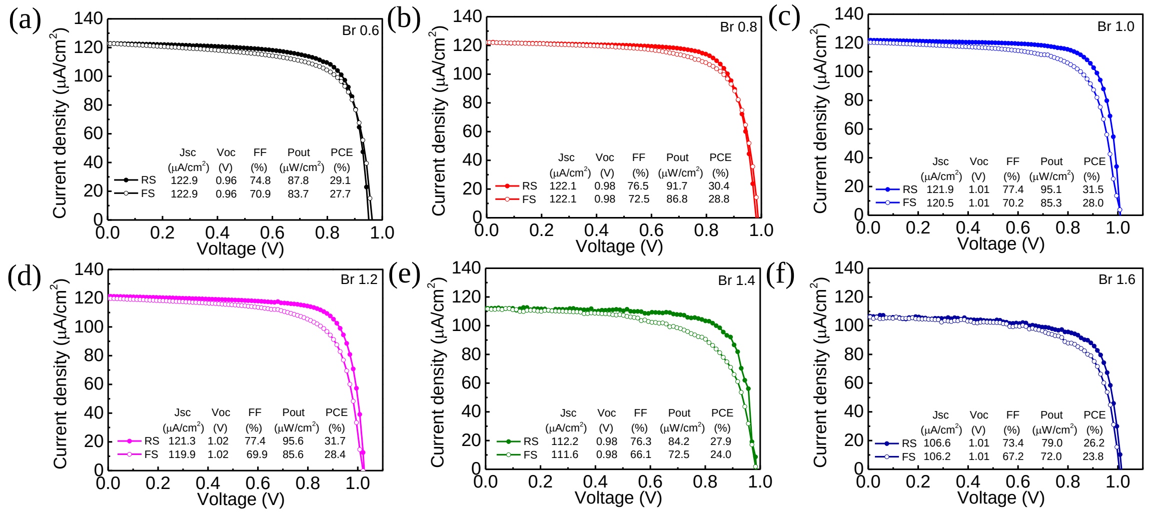

Figure S8. Typical *J-V* curves of perovskite IPVs with different Br contents.

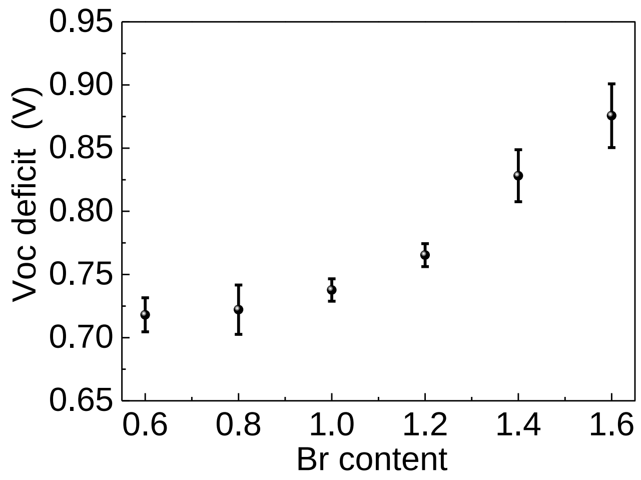

Figure S9. V_{OC} deficit statistics of the devices with different Br contents.

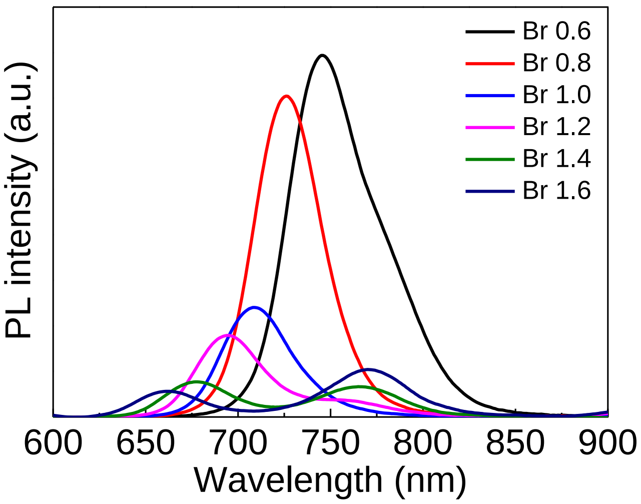

Figure S10. Steady PL spectra of perovskite thin films with different Br contents.

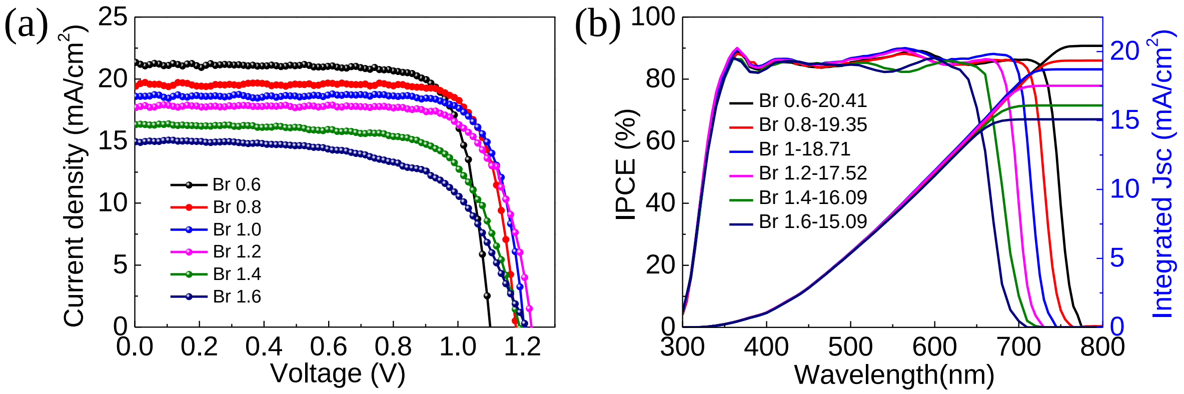

Figure S11. (a) The *J-V* curves measured under standard sunlight (AM1.5 G, 100 mW/cm^2) of perovskite solar cells with different Br contents. (b) EQE spectra and the integrated current density curves of corresponding devices.

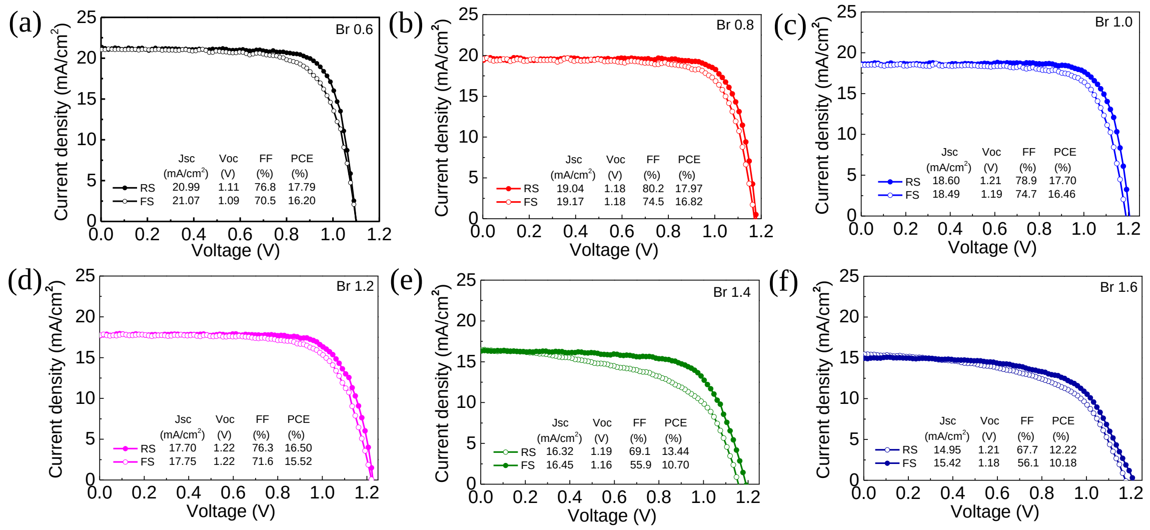

Figure S12. Typical *J-V* curves measured under standard sunlight of perovskite solar cells with different Br contents.

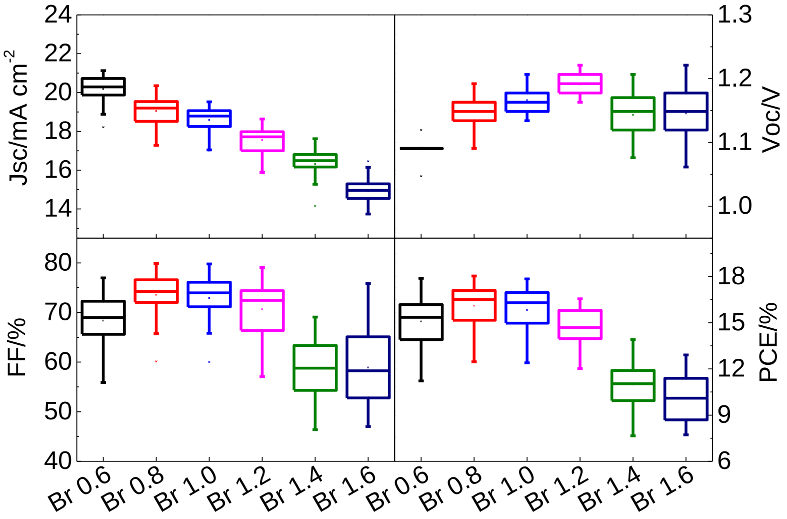

Figure S13. The statistics of parameters change of perovskite solar cells (devices number = 50) with varying Br contents.

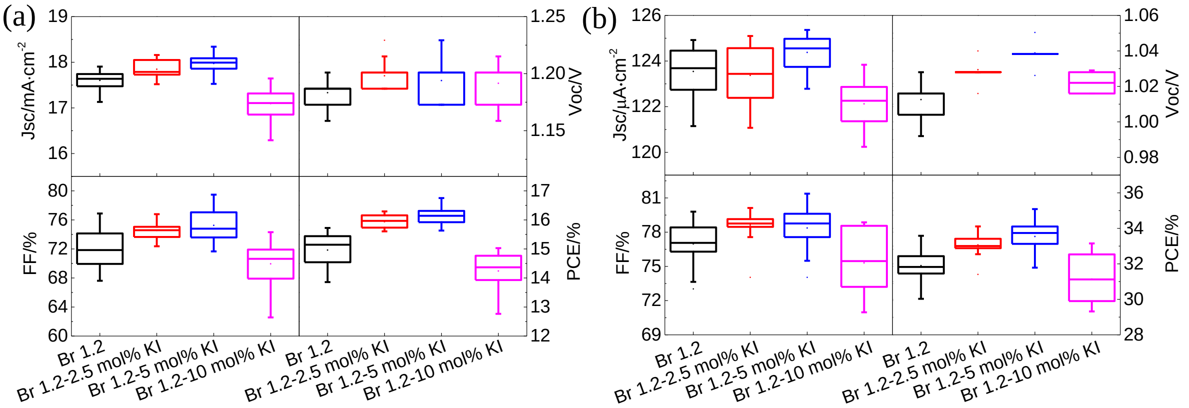

Figure S14. The statistics of parameters of perovskite photovoltaic cells with varying KI concentrations at (a) AM1.5 G and (b) 1000 lux 3000 K LED.

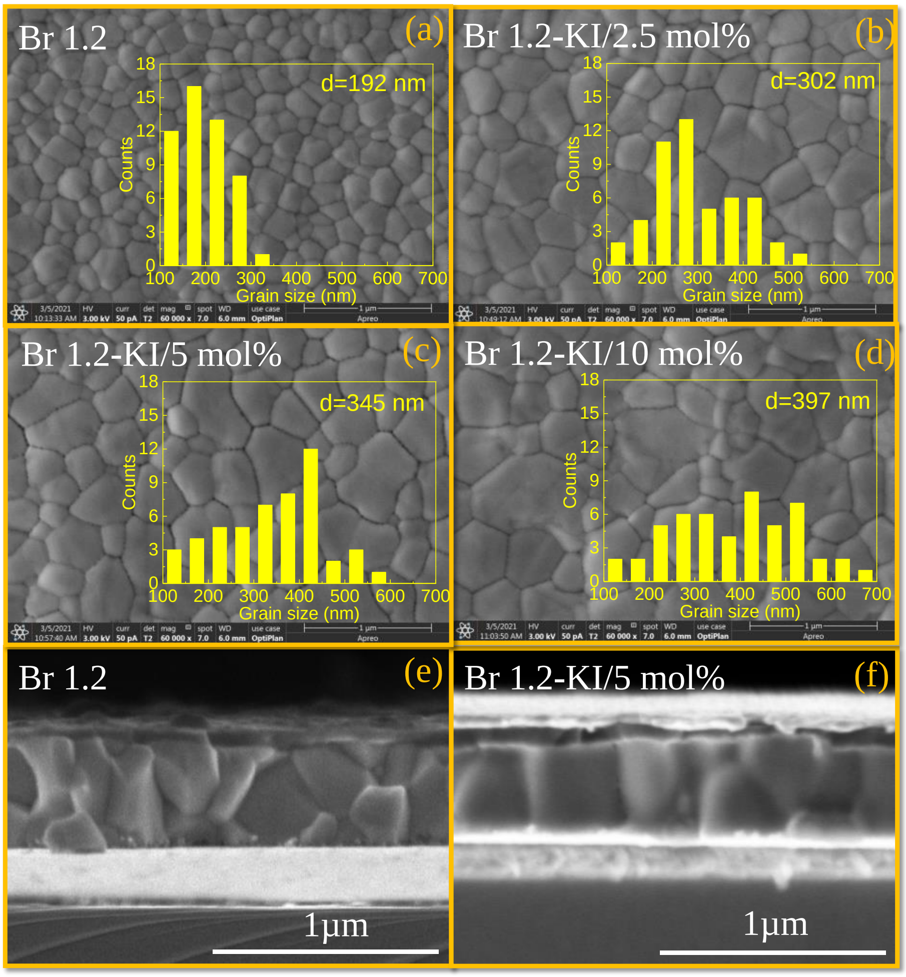

Figure S15. Surface and cross-sectional SEM images of the perovskite thin films with different amount of KI additions.

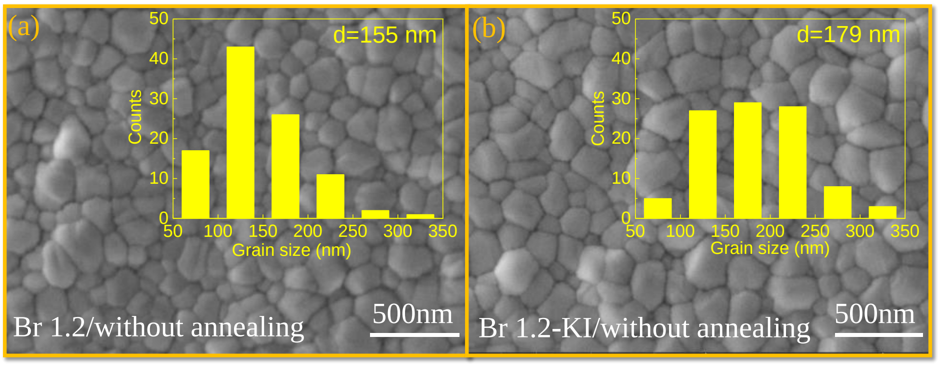

Figure S16. Top view SEM images of the perovskite thin films after anti-solvent process without annealing.

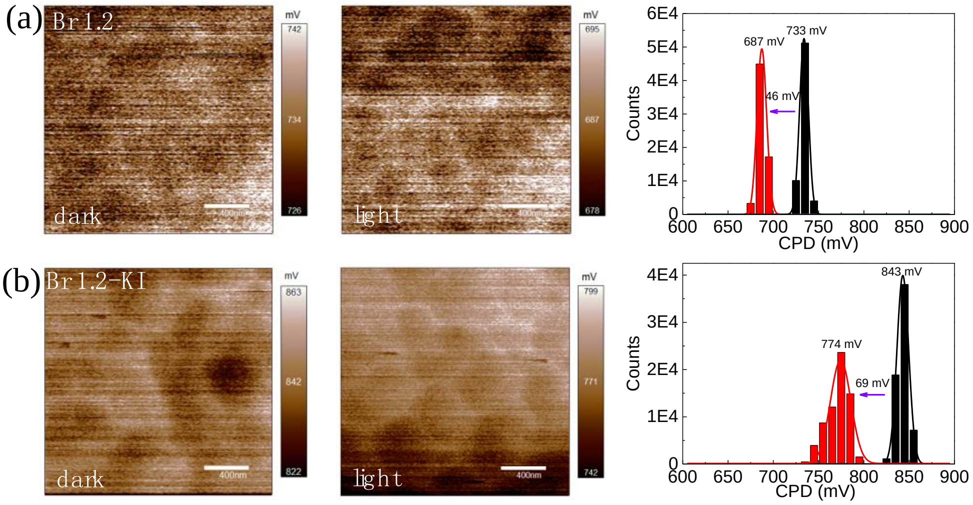

Figure S17. CPD distribution maps and the quantized values in dark and light illumination of (a) reference and (b) KI-treated samples.

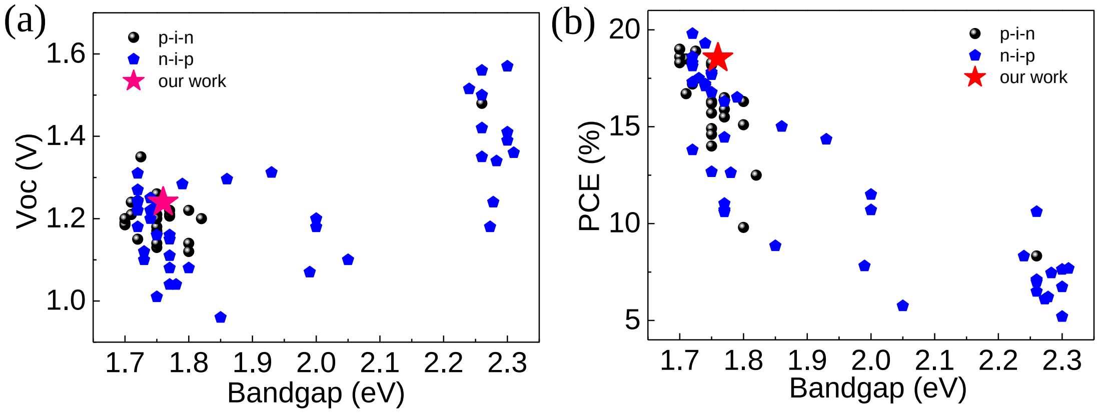

Figure S18. Statistics of V_{OC} and PCE as a function of reported wide-bandgap organic-inorganic perovskite solar cells.

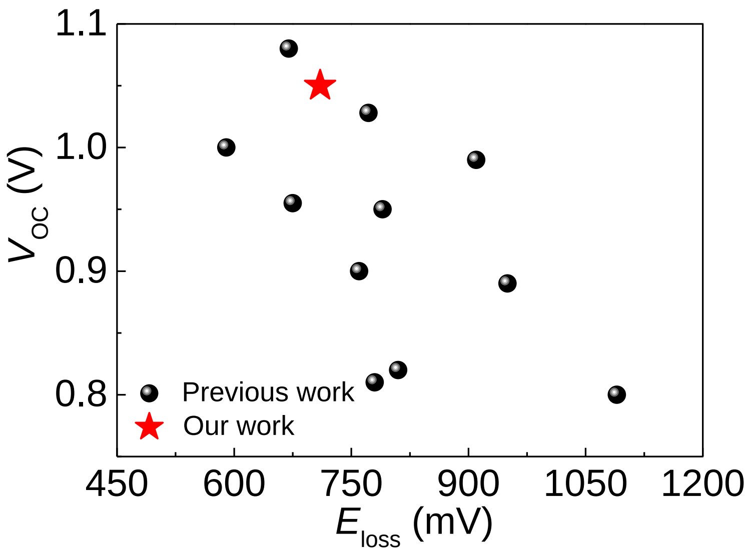

Figure S19. Statistic of V_{OC} as a function of E_{loss} reported perovskite IPVs at 1000 lux.

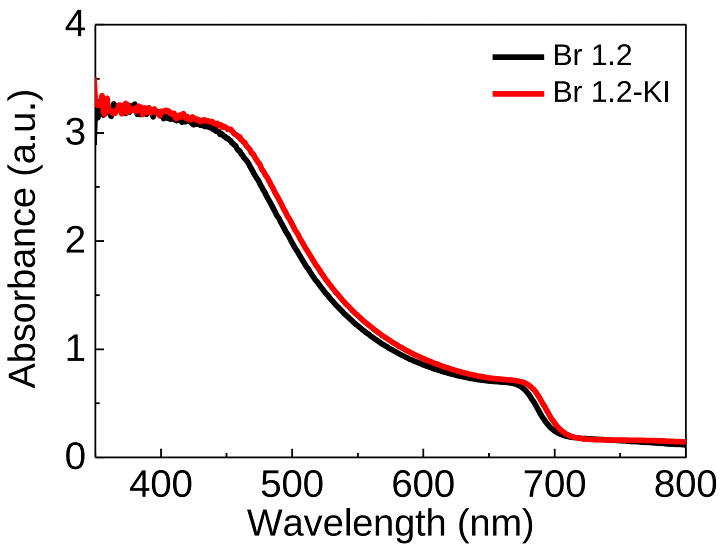

Figure S20. UV-vis absorption spectra of Br 1.2 and Br 1.2-KI thin films.

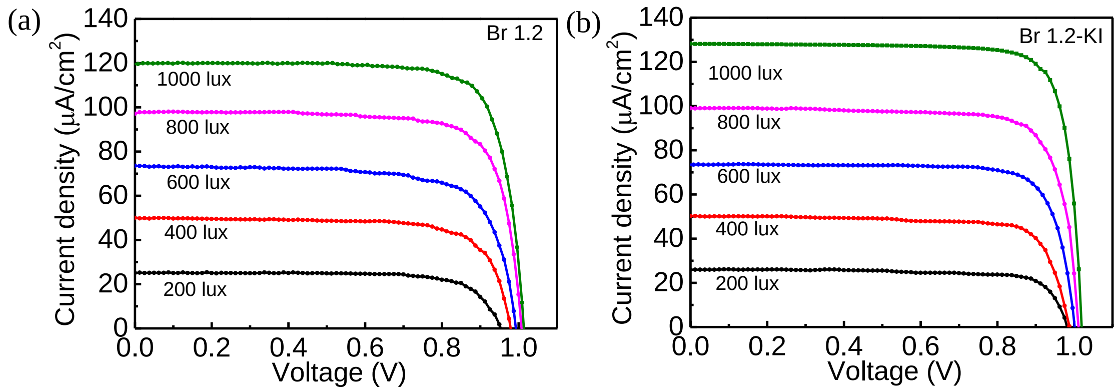

Figure S21. *J*-*V* curves of (a) Br 1.2 and (b) Br 1.2-KI devices at 200-1000 lux.

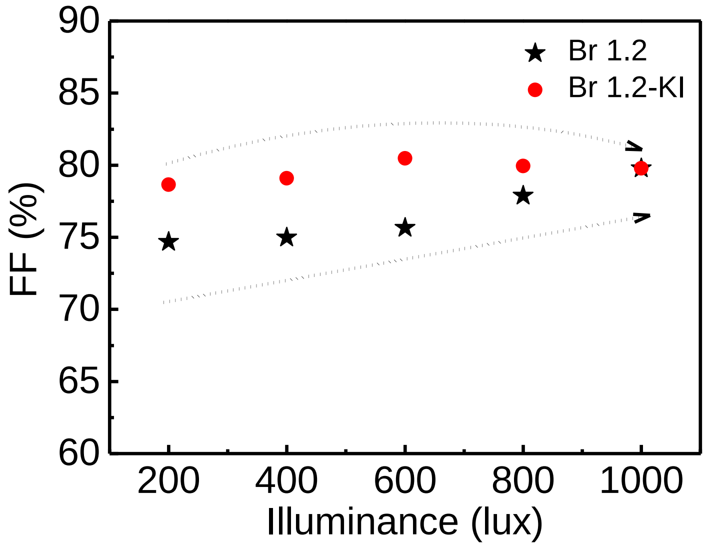

Figure S22. FF of Br 1.2 and Br 1.2-KI cells varies with illumination.

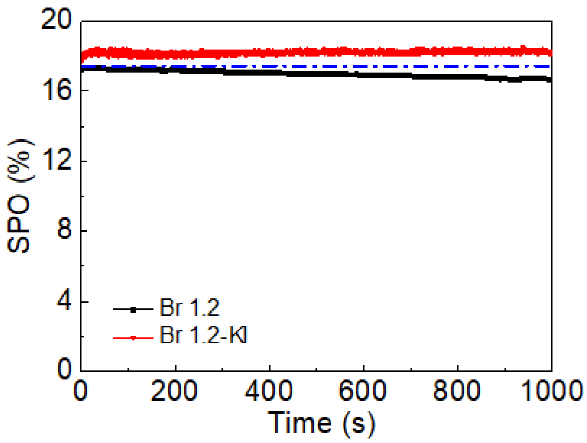

Figure S24. SPO measurement as a function of time under standard sunlight.

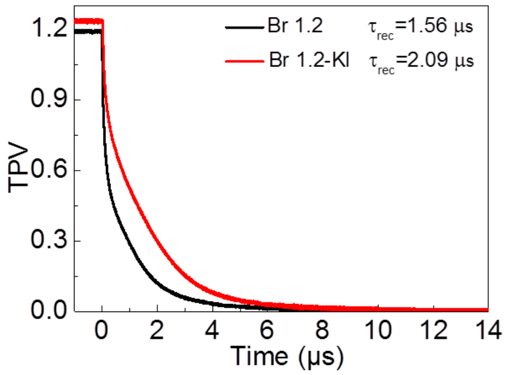

Figure S25. V_{OC} decay as a function of time.

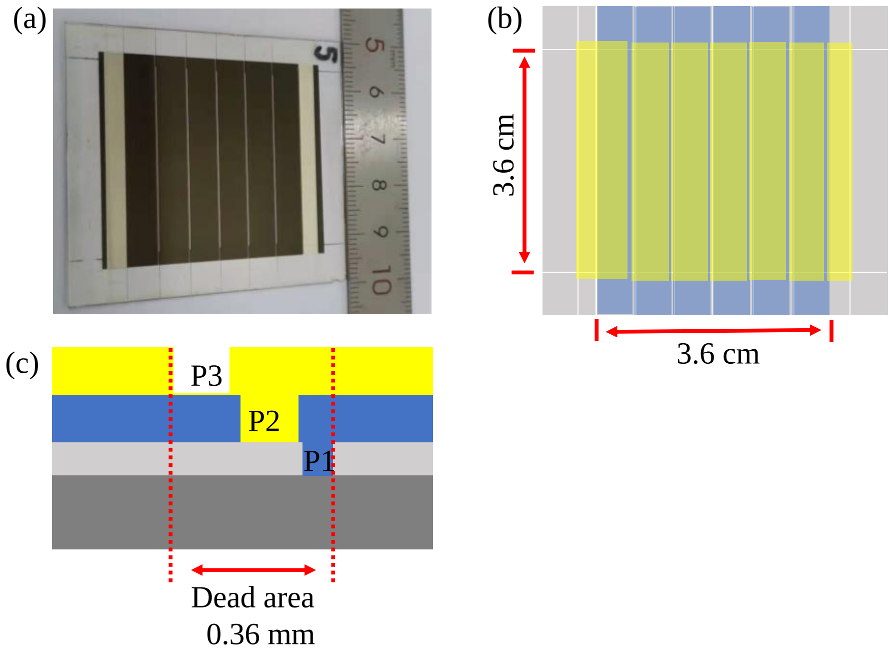

Figure S26. (a) Photograph of the perovskite module. (b) Schematic diagram of perovskite module with 6 subcells connected in series. (c) Illustration of the module interconnection.

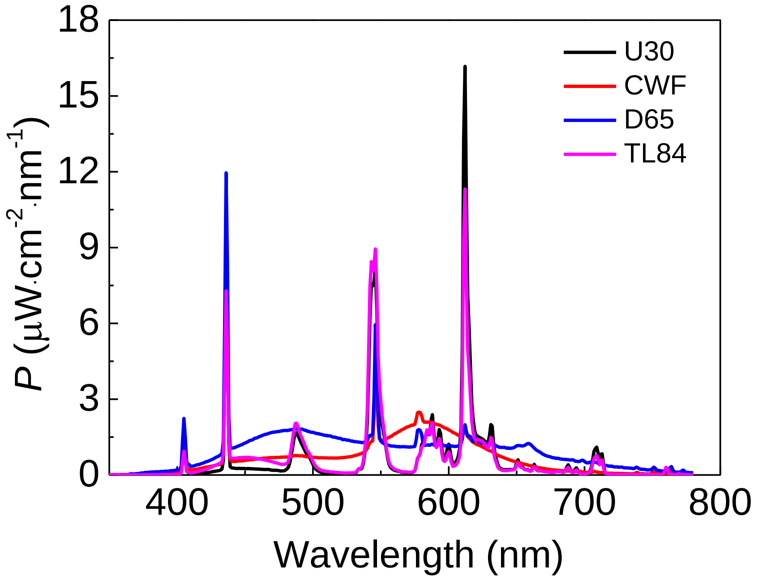

Figure S27. The emission spectra of U30, CWF, D65 and TL84 light source at 1000 lux.

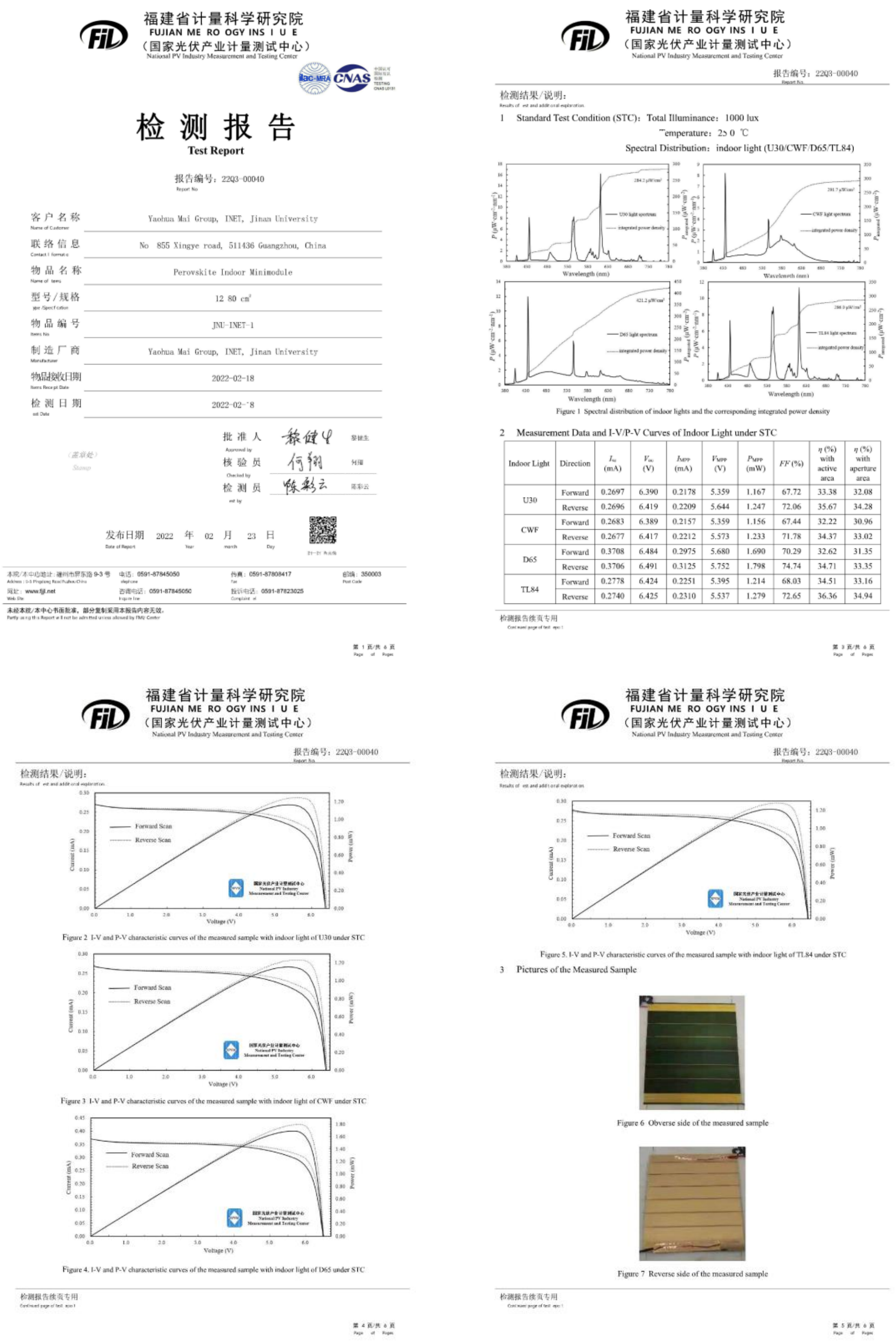
福建省计量科学研究院
FUJIAN METROLOGY INSTITUTE
（国家光伏产业计量测试中心）
National PV Industry Measurement and Testing Center

检 测 报 告
Test Report

报告编号：22Q3-00040

客户名称 Name of Customer: Yaohua Mai Group, INET, Jinan University
联络信息 Contact Information: No 855 Xingye road, 511436 Guangzhou, China
物品名称 Name of Items: Perovskite Indoor Minimodule
物品编号 Items No: JNU-INET-1
制造厂商 Manufacturer: Yaohua Mai Group, INET, Jinan University
物品接收日期 Items Receipt Date: 2022-02-18

批准人 Approved by
核验员 Checked by
检测员 Test by

发布日期 Date of Report: 2022 年 02 月 23 日

检测结果/说明：
1 Standard Test Condition (STC): Total Illuminance: 1000 lux
Spectral Distribution: indoor light (U30/CWF/D65/TL84)

Figure 1 Spectral distribution of indoor lights and the corresponding integrated power density

2 Measurement Data and I-V/P-V Curves of Indoor Light under STC

Indoor Light	Direction	I_{sc} (mA)	V_{oc} (V)	I_{MPP} (mA)	V_{MPP} (V)	P_{MPP} (mW)	FF (%)	η (%) with active area	η (%) with aperture area
U30	Forward	0.2697	6.390	0.2178	5.359	1.167	67.72	33.38	32.08
	Reverse	0.2696	6.419	0.2209	5.644	1.247	72.06	35.67	34.28
CWF	Forward	0.2683	6.389	0.2157	5.359	1.156	67.44	32.22	30.96
	Reverse	0.2677	6.417	0.2212	5.573	1.233	71.78	34.37	33.02
D65	Forward	0.3708	6.484	0.2975	5.680	1.690	70.29	32.62	31.35
	Reverse	0.3706	6.491	0.3125	5.752	1.798	74.74	34.71	33.35
TL84	Forward	0.2778	6.424	0.2251	5.395	1.214	68.03	34.51	33.16
	Reverse	0.2740	6.425	0.2310	5.537	1.279	72.65	36.36	34.94

Figure 2 I-V and P-V characteristic curves of the measured sample with indoor light of U30 under STC

Figure 3 I-V and P-V characteristic curves of the measured sample with indoor light of CWF under STC

Figure 4. I-V and P-V characteristic curves of the measured sample with indoor light of D65 under STC

Figure 5. I-V and P-V characteristic curves of the measured sample with indoor light of TL84 under STC

3 Pictures of the Measured Sample

Figure 6 Obverse side of the measured sample

Figure 7 Reverse side of the measured sample

Figure S28. Certificated results from accredited photovoltaic certification laboratory (Fujian Metrology Institute, National PV Industry Measurement and Testing Center).

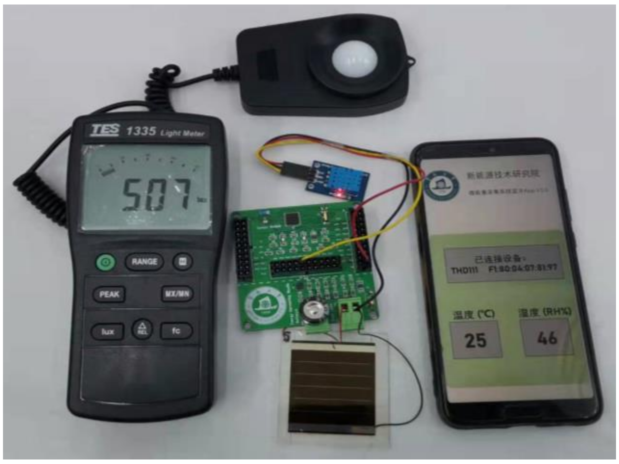

Figure S29. Photograph showing a perovskite photovoltaic module with 6 subcells connected in series powering an electrical circuit in ambient environment under 507 lux illumination.

Table S1. The band gaps of perovskite thin films with different Br contents.

Br content	Band gap (ev)
Br 0.6	1.65
Br 0.8	1.70
Br 1.0	1.74
Br 1.2	1.77
Br 1.4	1.82
Br 1.6	1.86

Table S2. Detailed performance parameters of Br 1.2 and Br 1.2-KI devices at 200-1000 lux.

Device type	Illuminance (lux)	$^{a}P_{in}$ (μW/cm^2)	*J*sc (μA/cm^2)	V_{OC} (V)	FF (%)	$^{b}P_{out}$ (μW/cm^2)	PCE (%)
Br 1.2	200	60.4	25.00	0.95	74.68	17.74	29.38
	400	120.8	49.76	0.98	74.98	36.38	30.13
	600	181.2	73.33	0.99	75.66	54.78	30.23
	800	241.5	97.05	1.00	77.58	75.29	31.18
	1000	301.9	119.59	1.01	78.59	94.59	31.33
Br 1.2-KI	200	60.4	25.74	0.98	78.65	19.73	32.68
	400	120.8	49.43	0.99	79.08	38.60	31.96
	600	181.2	73.48	1.01	80.46	59.66	32.93
	800	241.5	98.35	1.02	79.94	79.61	32.96
	1000	301.9	121.59	1.03	79.79	99.44	32.94

$^{a}P_{in}$: Power input $^{b}P_{out}$: power output

Table S3. The reported photovoltaic parameters of large area perovskite indoor photovoltaic cells and modules under indoor light.

type	Area (cm^2)	Illuminance (lux)	*J*sc (μA/cm^2)	V_{OC} (V)	FF	PCE (%)	Reference
Large area	5.44	1000	118.49	0.75	0.71	20.10	[1]
	4	1000	108.00	0.84	0.71	23.16	[2]
	4	1000	111.00	0.87	0.77	23.90	[3]
	2.25	1000				30.60	[4]
Module	4	1000				17.89	[5]
	12.96	1000	274.00	6.42	0.73	36.36	This work

Supplementary Note 1

Calculation of the SQ limits for artificial light sources

The calculation of the SQ limits in this manuscript follows a script published by Steve Byrnes,[6] which is primarily based on previous Python version.[7] A Jupyter notebook on GitHub is also provided to reveal the details of the calculations in this study.[8]

The spectra of the two artificial light sources were measured using an Everfine spectral illuminometer; the four spectra are also available on GitHub.[8]

Supplementary Note 2

Indoor light source power density calculation

E_v=683 $\int E_e(\lambda)V(\lambda)d\lambda$

E_e=$\int E_e(\lambda)d\lambda$

$$T = \frac{\int E_e(\lambda)d\lambda}{683\int E_e(\lambda)V(\lambda)d\lambda}$$

$$E_e(\lambda) \ = \alpha * ee(\lambda)$$

$$T = \frac{\int ee(\lambda)d\lambda}{683\int ee(\lambda)V(\lambda)d\lambda}$$

E_v= Integral illumination.

V(λ): Standard Luminosity function.

$E_{e:}$ Integral irradiance.

E_e (λ): light intensity per nm, W/cm^2/nm. It can’t be measured directly.

T: conversion coefficient.

α: proportionality factor.

ee(λ): relative value of E_e (λ).

The power density under the corresponding illumination (lux) was calculated according to the shape of the spectrum.

References

[1] C. Y. Chen, J. H. Chang, K. M. Chiang, H. L. Lin, S. Y. Hsiao, H. W. Lin, *Adv. Funct. Mater.* **2015**, 25, 7064-7070.

[2] M. Li, C. Zhao, Z. K. Wang, C. C. Zhang, H. K. Lee, A. Pockett, J. Barbé, W. C. Tsoi, Y. G. Yang, M. J. Carnie, *Adv. Energy Mater.* **2018**, 8, 1801509.

[3] C.-Y. Chen, W.-H. Lee, S.-Y. Hsiao, W.-L. Tsai, L. Yang, H.-L. Lin, H.-J. Chou, H.-W. Lin, *J. Mater. Chem. A* **2019**, 7, 3612-3617.

[4] R. Cheng, C. C. Chung, H. Zhang, F. Liu, W. T. Wang, Z. Zhou, S. Wang, A. B. Djurišić, S. P. Feng, *Adv. Energy Mater.* **2019**, 9, 1901980.

[5] M. J. Wu, C. C. Kuo, L. S. Jhuang, P. H. Chen, Y. F. Lai, F. C. Chen, *Adv. Energy Mater.* **2019**, 9, 1901863.

[6] http://sjbyrnes.com/sq.pdf

[7] https://github.com/evcka/sq-limit

[8] https://github.com/zsshhxxttxs/Calculation-the-limit-efficiency-of-solar-cells-under-artificial-light-sources-#readme
